# Systematic synthesis of a 6-component organic-salt alloy of naftopidil, and pentanary, quaternary and ternary multicomponent crystals

**DOI:** 10.1107/S2052252518014057

**Published:** 2018-10-24

**Authors:** Rambabu Dandela, Srinu Tothadi, Udaya Kiran Marelli, Ashwini Nangia

**Affiliations:** a Organic Chemistry Division, CSIR-National Chemical Laboratory, Dr. Homi Bhabha Road, Pune, Maharashtra 411 008, India; b Central NMR Facility, CSIR-National Chemical Laboratory, Dr. Homi Bhabha Road, Pune, Maharashtra 411 008, India; c School of Chemistry, University of Hyderabad, Prof. C. R. Rao Road, Gachibowli, Hyderabad, Telangana 500 046, India

**Keywords:** active pharmaceutical ingredients, crystal engineering, naftopidil, multicomponent solids, 6-component crystals, organic-salt alloys

## Abstract

The single-crystal X-ray structure of a 6-component organic-salt alloy (hexanary) of naftopidil (1) (an active pharmaceutical ingredient) with benzoic acid (2) and four different hydroxy-substituted benzoic acids, *i.e.* salicylic acid (3), 2,3-di­hydroxybenzoic acid (4), 2,4-di­hydroxybenzoic acid (5) and 2,6-di­hydroxybenzoic acid (6), is reported. The hexanary assembly originates from the observation that the binary salts of naftopidil with the above acids are isostructural. In addition to the 6-component solid, we also describe five 5-component, ten 4-component, and ten 3-component organic-salt alloys of naftopidil (1) with carboxylic acids (2)–(6). These alloys were obtained from different combinations of the acids with the drug. The synthetic design of the multicomponent organic alloys is based on the rationale of geometrical factors (shape and size) and chemical interactions (hydrogen bonds). The common supramolecular synthon in all these crystal structures was the cyclic N^+^—H⋯O^−^ and O—H⋯O hydrogen-bonded motif of (9) graph set between the 2-hy­droxyammonium group of naftopidil and the carboxyl­ate anion. This ionic synthon is strong and robust, directing the isostructural assembly of naftopidil with up to five different carboxylic acids in the crystal structure together with the lower-level multicomponent adducts. Solution crystallization by slow evaporation provided the multicomponent organic salts and alloys which were characterized by a combination of single-crystal X-ray diffraction, powder X-ray diffraction, NMR and differential scanning calorimetry techniques.

## Abstract 2

A 6-component organic-salt alloy of naftopidil with hy­droxy-substituted benzoic acids is designed based on a recurrent supramolecular synthon and the replacement of coformer acids in the crystal lattice. Isostructurality of five binary salts leads to the crystallization of multicomponent salt alloys. One 6-component crystal, five 5-component, ten 4-component and ten 3-component organic-salt alloys of naftopidil with carboxylic acids are reported.

## 1. Introduction   

Inorganic solid solutions are well known, widely prepared and extensively used in day-to-day life (Kumar, 2008 ▸). Fine tuning the ratio of components in inorganic alloys is relatively easy because of the spherical nature of atoms and ions involved in the multicomponent crystal structures. On the other hand, synthesis of organic alloys is challenging due to the different sizes, shapes and functional groups of the molecules. Hence, organic solid solutions are not so extensively studied in the literature. Organic alloys (Khoj *et al.*, 2017 ▸; Lusi *et al.*, 2015 ▸; Thomas *et al.*, 2015 ▸) are different from organic salts and cocrystals; they are crystalline materials in which at least two different molecules are present, but not in a fixed stoichiometric ratio, whereas organic salts and cocrystals have a definite molecular stoichiometry. As a corollary, organic-salt alloys are characterized by the complete transfer of a proton from one molecule to another, with the components being present in variable stoichiometries in the ionic supramolecular structure (Mittapalli *et al.*, 2015 ▸; Reddy *et al.*, 2011 ▸; Suresh *et al.*, 2017 ▸; Allu *et al.*, 2017 ▸; Bond, 2007 ▸).

The rational design and synthesis of multicomponent pharmaceutical crystals is extremely challenging as the number of components increases, representing a significant advance for academia with immediate utility for the pharmaceutical industry (Almarsson *et al.*, 2004 ▸; Duggirala *et al.*, 2016 ▸; Bolla & Nangia, 2016 ▸; Thipparaboina *et al.*, 2016 ▸). Crystallization is inherently a purification technique, and the outcome is mostly separation of the single-component crystals rather than a multicomponent assembly. Inclusion of more than three different molecules in the cocrystal becomes increasingly difficult (Tothadi & Desiraju, 2013 ▸). For instance, if one wishes to synthesize ternary cocrystals of A, B and C, then there is always a possibility to form ABC, but AB, AC, and solvates, hydrates and also polymorphs of the individual components are complicating side products. Hence, to reach the target multicomponent crystalline material is not always straightforward. The systematic design of multicomponent molecular crystals requires a proper understanding and optimization of the geometrical factors (molecular shape and size) and chemical interactions, such as hydrogen-bond donors and acceptors, *i.e.* supramolecular synthons (Desiraju, 1995 ▸; Tothadi *et al.*, 2011 ▸). Multicomponent cocrystals have considerable importance in the pharmaceutical industry for the fine tuning of the physicochemical and pharmacokinetic properties of drugs such as solubility, dissolution, stability and bioavailability to name a few (Sanphui *et al.*, 2012 ▸; Babu *et al.*, 2012 ▸; Trask *et al.*, 2005 ▸; Cherukuvada *et al.*, 2011 ▸; Smith *et al.*, 2011 ▸; Wouters & Quéré, 2012 ▸; Walsh *et al.*, 2003 ▸). Crystal engineering (Desiraju, 1989 ▸) has reached the stage wherein from two component pharmaceutical cocrystals at the beginning of this millennium, (Almarsson *et al.*, 2004 ▸), the recent record has become five components (Mir *et al.*, 2016 ▸; Dubey *et al.*, 2016 ▸) in 2016 and a 6-component organic adduct (Paul *et al.*, 2018 ▸) has been reported recently while this manuscript was in the final stages. Furthermore, this is the first report of six different molecular components for an organic-salt alloy of an active pharmaceutical ingredient solved and refined by single-crystal X-ray diffraction. Binary, ternary and even quaternary cocrystals have been reported by several research groups during the past three decades (Reddy *et al.*, 1994 ▸; Thakur *et al.*, 2010 ▸; Sada *et al.*, 2005 ▸; Hasell *et al.*, 2012 ▸; Tothadi *et al.*, 2017 ▸; Dabros *et al.*, 2007 ▸; Allu *et al.*, 2017 ▸). The bar was raised by Desiraju with the synthesis of 4- and 5-component molecular assemblies in 2016 (Mir *et al.*, 2016 ▸; Dubey *et al.*, 2016 ▸), and now a 6-component molecular solid (Paul *et al.*, 2018 ▸) has been achieved by combining the design strategies of cocrystals and solid solution. They upgraded the initial 4-component synthetic dead-end cocrystal to a 6-component solid solution by incorporating molecules of a similar shape/size. In addition to the challenge of synthetic design, geometric and chemical matching, and supramolecular assembly of multicomponent organic crystals with six components, there are crystal structure refinement difficulties caused by overlap of electron-density maps of molecules having similar size/placement of functional groups, and sometimes disorder in the cocrystal (Tothadi & Desiraju, 2013 ▸; Thakur *et al.*, 2010 ▸; Hasell *et al.*, 2012 ▸; Tothadi *et al.*, 2017 ▸). Chemical factors start to taper off for 4-component crystals because weaker interactions provide only limited enthalpic advantage to molecular assembly in the cocrystal (Mir *et al.*, 2016 ▸; Dubey *et al.*, 2016 ▸). In this communication, we report the build-up of six different molecules/ions in an organic-salt alloy along with the lower 5-, 4- and 3-component structures based on a single robust supramolecular synthon. All in all, 25 multicomponent single-crystal structures, which include 20 salt alloys (one hexanary, three pentanary, eight quaternary and eight ternary) and five binary salts contain the same ionic 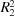
(9) supramolecular synthon. An additional six alloys were characterized by powder X-ray diffraction (PXRD), NMR and differential scanning calorimetry (DSC) techniques in the absence of suitable single crystals (two each of ternary, quaternary and pentanary). Conceptually, our approach to a 6-component assembly based on a strong ionic synthon and isomeric carboxylic-acid replacement complements the Desiraju template (Paul *et al.*, 2018 ▸) based on crystallographic inequivalences and halogen exchange.

## 2. Experimental   

Naftopidil (in racemic form) was provided by Trimax Bio Sciences Ltd (India). The coformer and solvents (>99% purity) were purchased from TCI chemicals (Pune, India).

### 2.1. X-ray crystallography   

A few single crystals obtained after crystallization were mounted on a Super Nova Dual Source X-ray Diffractometer system (Agilent Technologies) equipped with a CCD area detector. The X-ray generator was operated at 50 kV and 0.8 mA to generate Mo *K*α radiation (λ = 0.71073 Å) and Cu *K*α radiation (λ = 1.54178 Å) at 298 (2) K. Data reduction was performed using *CrysAlisPro* software (version 171.33.55, Agilent, 2010 ▸). The remaining crystals were mounted on a Bruker SMART APEX II single-crystal X-ray CCD diffractometer having graphite-monochromated Mo *K*α radiation (λ = 0.71073 Å) at 100 and 293 K. The X-ray generator was operated at 50 kV and 30 mA. X-ray data acquisition was monitored by the *APEX2* program suite. The data were corrected for Lorentz–polarization and absorption effects using *SAINT* and *SADABS* which are integral parts of the *APEX2* package (Bruker, 2006 ▸). The structures were solved by direct methods and refined by full-matrix least squares based on *F* ^2^ and using *SHELXL* (Sheldrick, 2017 ▸). Crystal structures were refined using *OLEX* software (version 2-1.0, Dolomanov *et al.*, 2009 ▸) (Tables 1 ▸ and S2 of the supporting information). Anisotropic refinement was performed for all non-hydrogen atoms. Most hydrogen atoms attached to heteroatoms were located using the difference Fourier map and some O—H hydrogen atoms were geometrically fixed using the HFIX command in *SHELXL*. All hydrogen atoms were refined isotropically. The structures were examined using the *ADSYM* subroutine of *PLATON* (Spek, 2005 ▸) to ensure that no additional symmetry could be applied to the models. *Mercury* software was used to prepare packing diagrams and molecular interactions. CIFs (CCDC references 1817125, 1817126, 1817127, 1817128, 1817129, 1817130, 1817146, 1817147, 1817148, 1817149, 1817150, 1817151, 1817158, 1817159, 1817160, 1817161, 1817162, 1817163, 1817164, 1817165, 1817166, 1817167, 1817168, 1817169, 1817170, 1840048) are available at https://www.ccdc.cam.ac.uk/data or as part of the supporting information.

*Refinement of organic-salt alloy crystal data:* first, all non-hydrogen atoms were refined isotropically followed by anisotropic refinement. Substituted oxygen atoms of the acids (coformers) showed unusual ellipsoid electron density caused by multiple atoms in the envelope; free refinement was performed on the atoms to find out the exact ratio of the corresponding acids. Then the organic-salt alloy composition of acid ratios was fixed by minimizing to an acceptable *R* factor. Finally, anisotropic refinement was performed. To show the variable occupancy, PART, EADP and EXYZ commands were used in the .ins file. The acids of all salt alloys showed variable occupancy and disorder in the molecules which appeared as an unusual C—O—H geometry and short inter-distances: *D*—H⋯H—*X*. Reasonable chemical conclusions were derived from the crystallographic data.

### 2.2. Powder X-ray diffraction   

All crystalline material PXRD data were recorded on a Rigaku, MicroMax-007HF using a high-intensity microfocus rotating-anode X-ray generator and a Cu *K*α (λ = 1.54178 Å) radiation source with an Ni filter. Data collection was carried out using an aluminium holder at a scan speed of 1° min^−1^ and a step size of 0.02°. All crystalline samples were scanned over range of 2θ = 2–40° and the corresponding data were collected using *Control Win* software.

### 2.3. Thermal analysis   

DSC was performed on a Mettler-Toledo DSC 822e module, (Mettler-Toledo, Columbus, OH). Samples were placed in crimped but vented aluminium pans for DSC experiments; the typical sample size was 3–5 mg. The temperature range for the heating curves was 30–300°C, and the sample was heated at a rate of 5°C min^−1^. Samples were purged in a stream of dry nitro­gen flowing at 80 ml min^−1^.

### 2.4. NMR   

All the one-dimensional ^1^H NMR experiments for the individual components, alloy crystals and physical mixtures were recorded in DMSO-*d*_6_ at 298 K on a Bruker Avance 500 MHz NMR spectrometer equipped with a BBO probe. Two-dimensional NMR (^1^H–^1^H DQF-COSY, TOCSY, ROESY, ^13^C–^1^H HSQC and HMBC) experiments for the 6-component alloy crystal were recorded in DMSO-*d*_6_ at 298 K on a Bruker Avance III HD 700 MHz NMR spectrometer equipped with a BBO probe. 2k complex points were acquired in direct dimension of all two-dimensional experiments except for HSQC, where 1k complex points were acquired. While 320 increments were recorded in the indirect dimension of COSY, TOCSY and HSQC, 512 and 576 increments were recorded for ROESY and HMBC, respectively.

Chemical-shift assignment and identification of all the components in the 6-component alloy crystal was achieved by detailed analysis of chemical shifts, resonance integral values, *J*-multiplicity and coupling constants in one-dimensional-^1^H experiments and further confirmed by chemical shift correlations from two-dimensional experiments. The ratio of the individual components in the 6-component alloy crystal was determined from the ratio of the integrals of the resonances in its one-dimensional-^1^H experiment, which was acquired with a long d1 time of 10 s.

Identification of the rest of the alloy crystals (123, 1456, 12345, 12346, 12356, 12456 and 13456) was done by analysis of chemical shifts, resonance integral values, *J*-multiplicity and coupling constants in corresponding one-dimensional-^1^H experiments and further correlation to the chemical shifts in the 6-component alloy crystal.

### 2.5. Cambridge Structural Database analysis   

The 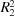
(9) synthon was drawn using *ConQest* and the intermolecular distance was taken as the distance within the sum of the van der Waals radii + 0.0 Å.

## 3. Results and discussion   

**Figure 1:**
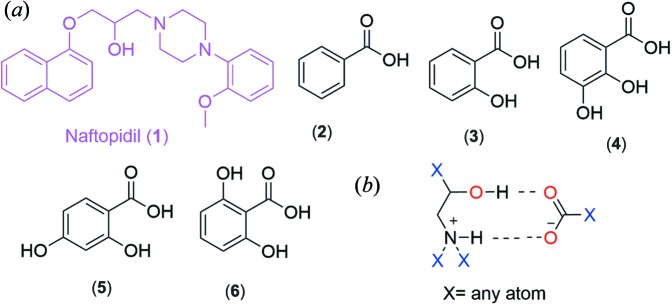
(*a*) Naftopidil (1) and coformers benzoic acid (2), 2-hy­droxy­benzoic acid, (3) 2,3-di­hydroxy­benzoic acid (4), 2,4-di­hydroxy­benzoic acid (5) and 2,6-di­hydroxy­benzoic acid (6) used for cocrystallization; and (*b*) the ionic synthon of the 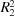
(9) graph set that is observed in all single-crystal structures (salts). In the synthon, complete proton transfer produces an N^+^—H⋯O^−^ and O—H⋯O hydrogen-bonded cyclic motif.

**Figure 2:**
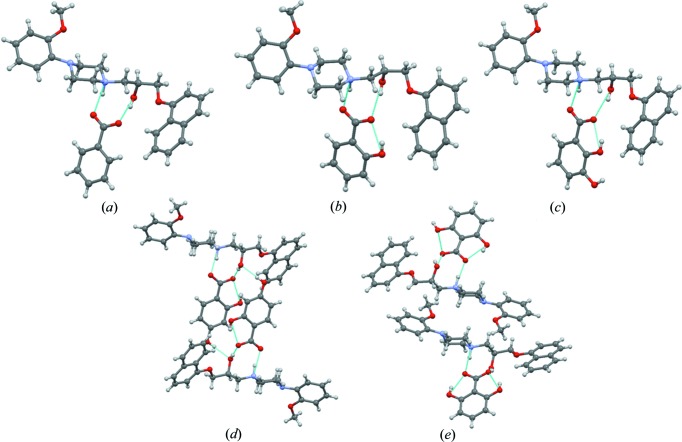
Binary salts of naftopidil (1) with coformers (2)–(6): (*a*) (12), (*b*) (13), (*c*) (14), (*d*) (15) and (*e*) (16).

**Figure 3:**
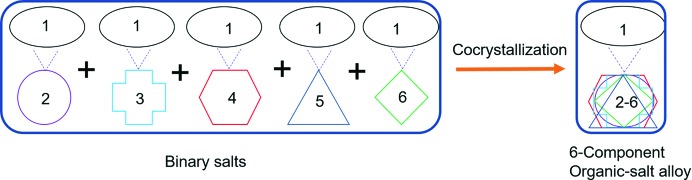
Synthesis of multicomponent organic-salt alloys: the first five binary salts [(12), (13), (14), (15) and (16)] were synthesized and their structures analyzed. All binary salts are isostructural. Similarly ternary, quaternary and pentanary crystal alloys were crystallized. Cocrystallization of naftopidil (1) (1.0 mmol) with benzoic acid (2), salicylic acid (3), 2,3-di­hydroxy­benzoic acid (4), 2,4-di­hydroxy­benzoic acid (5) and 2,6-di­hydroxy­benzoic acid (6) (0.2 mmol of each coformer) resulted in a 6-component organic-salt alloy (hexanary alloy).

**Figure 4:**
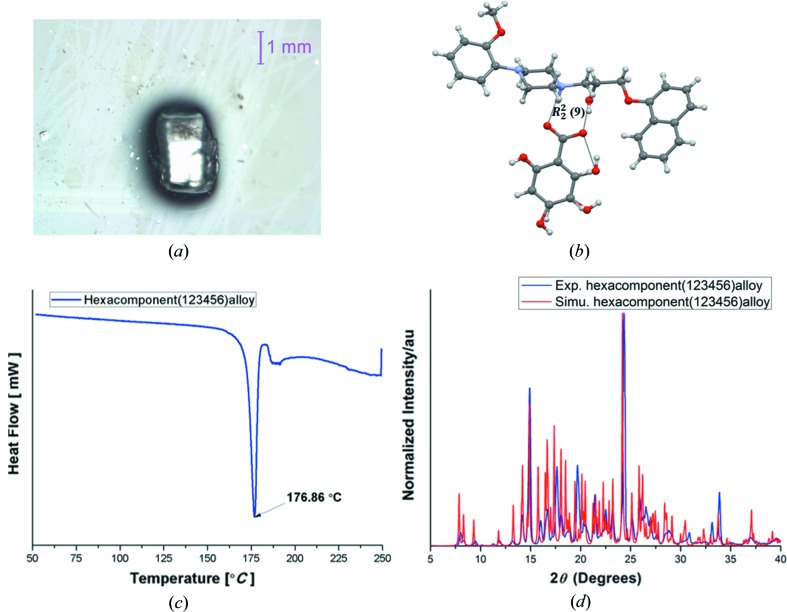
Hexacomponent (123456) alloy: (*a*) single crystal, (*b*) single-crystal structure showing one full molecule of naftopidil (1) and five acids with a site occupancy factor of 0.12 benzoic acid (2), 0.27 2-hydroxybenzoic acid (3), 0.14 2,3-di­hydroxybenzoic acid (4), 0.20 2,4-di­hydroxybenzoic acid (5) and 0.27 2,6-di­hydroxybenzoic acid (6). The binary salts form an 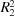
(9) synthon which is also present in the hexacomponent (123456) alloy. (*c*) DSC endotherm, showing a single melting point at 176°C which is different from the melting point of the single components as well as the binary salts. (*d*) PXRD shows bulk-phase purity experimental PXRD pattern compared with the calculated powder diffraction line pattern from the crystal structure.

Naftopidil (Xu *et al.*, 2017 ▸) is a selective α1-adrenergic receptor antagonist alpha-blocker antihypertensive drug. In the Biopharmaceutical Classification System (BCS) it is classified as a class IV drug of poor aqueous solubility and poor permeability (Xu *et al.*, 2017 ▸; Blume & Schug, 1999 ▸; Chavda *et al.*, 2010 ▸; Tsume *et al.*, 2014 ▸). There are no reports in the literature on either salts or cocrystals of naftopidil; therefore, we aimed to prepare new solid forms of naftopidil as part of a cocrystal screen. Five 1:1 binary salts of naftopidil (1) with the hydroxy-substituted benzoic acid series were prepared, with benzoic acid (2), 2-hydroxybenzoic acid (3), 2,3-di­hydroxybenzoic acid (4), 2,4-di­hydroxybenzoic acid (5) and 2,6-di­hydroxybenzoic acid (6) (Fig. 1 ▸). A 1:1 equimolar ratio of the drug and the coformer acid were taken in a mortar-pestle and ground for 15 min after the addition of 2–3 drops of solvent (MeOH). The ground product was added to MeOH (5 ml) and set aside for slow evaporation (a detailed experimental procedure is given in Table S1 of the supporting information). Good-quality single crystals (salts) were obtained from MeOH solution in a week, which were solved by single-crystal X-ray diffraction (Table 1 ▸). Interestingly, the five binary salts are isostructural (Table 1 ▸) and assemble *via* the 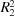
(9) motif where proton transfer from the coformer acid to the drug (1) and an N^+^−H···O^−^ plus O−H···O hydrogen bond is noted (Fig. 2 ▸). The novel salts of naftopidil were further characterized by PXRD (Fig. S6*a*) and DSC (Fig. S1). In the Cambridge Structural Database (CSD), there are 130 structures with the 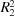
(9) motif [Fig. 1 ▸(*b*), Table S4]. The recurrence of the 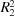
(9) synthon directs isostructurality, similar intermolecular interactions and crystal packing. The crystal structures of the individual benzoic acids are quite different from each other (analyzed from the CSD), highlighting the significance of the cyclic synthon 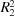
(9) in directing the multi-component structures. This observation suggested the synthesis of higher order multicomponent (3-, 4-, 5-component) salts or organic alloys (Fig. 3 ▸). To test the robustness of the 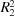
(9) synthon in this family, the salt of naftopidil (1) with 2,4,6-tri­hydroxybenzoic acid (as a surrogate of multiple hy­droxybenzoic acids occupying the same site) was prepared, and interestingly it also shows a similar crystal structure compared with the di­hydroxybenzoic acid binary salts (Fig. S44). This observation added confidence to the next experiments on higher-component assemblies. By considering the same supramolecular synthon (hydrogen bonds) as well as similar molecular size of the acids, and isostructurality of the binary crystal structures [Table 1 ▸, all crystallized in the space group *P*2_1_/*c* except (16) (*P*2_1_)], a 6-component organic-salt alloy was synthesized. Naftopidil (1) 1 mmol, and 0.2 mmol of each of the five benzoic acids [(2), (3), (4), (5), (6); sum up to 1 mmol] were added to 5 ml MeOH and kept at ambient temperature. Good-quality single crystals suitable for X-ray diffraction were obtained in a week (Fig. 4 ▸ *a*). In the alloy crystal which diffracted in *P*2_1_/*c* space group, there is one full molecule of naftopidil (1) and different ratios of acids (variable occupancy) depending on the size and hydrogen-bonding groups in the coformer. The site-occupancy factors (SOF) in the hexanary alloy are: naftopidil (1) 1.0, benzoic acid (2) 0.12, 2-hydroxybenzoic acid (3) 0.27, 2,3-di­hydroxybenzoic acid (4) 0.14, 2,4-di­hydroxybenzoic acid (5) 0.20 and 2,6-di­hydroxybenzoic acid (6) 0.27 (Fig. 4 ▸ *b*). The SOF values of the individual acids were derived from the free refinement of respective carbon- or oxygen-atom electron-density maps to reach the lowest *R* factor (see Section 2 and Table S3). The correctness of the solution was confirmed by an overlay of the experimental PXRD of the 6-component alloy with the calculated powder X-ray line pattern from the crystal structure (Fig. 4 ▸ *d*). Similar to the binary salts, the carb­oxy­lic acid proton is completely transferred to the N atom of (1) together with the O—H⋯O hydrogen bond of 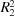
(9) in the hexanary alloy (123456). By applying a similar procedure, 25 organic-salt alloys of pentanary, quaternary and ternary composition (solid solutions) were synthesized to confirm the robustness of the strategy for multicomponent organic-salt alloys anchored by the 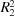
(9) synthon. The theoretical combinations will yield five pentanary, ten quaternary and ten ternary component crystals of naftopidil (1). For the synthesis of 5-component organic alloys, 1 mmol of (1) and 0.25 mmol of four different acids were used for cocrystallization (Table 2 ▸); 4-component organic alloys were synthesised using 1 mmol of (1) and 0.33 mmol each of the acids; and 3-component organic alloys were synthesised using 1 mmol of (1) and 0.5 mmol of the corresponding acids.

**Figure 5:**
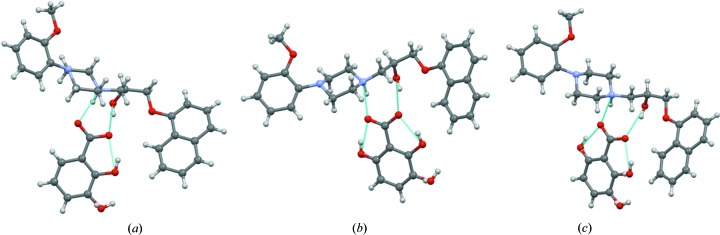
(*a*) Ternary (134) (*b*) quaternary (1346) and (*c*) pentanary (12346) organic-salt alloys. In the ternary (134) alloy, 2-hy­droxybenzoic acid and 2,3-di­hydroxybenzoic acid occupy the same site but with a variable occupancy of 0.49 and 0.51. Similarly, in the quaternary (1346) alloy the site occupancy of 2-hydroxybenzoic acid, 2,3-di­hydroxybenzoic acid and 2,6-di­hydroxybenzoic acid is 0.19, 0.43 and 0.38, respectively. Likewise, in pentanary (12346) alloy, the site occupancy of benzoic acid, 2-hydroxybenzoic acid, 2,3-di­hydroxybenzoic acid and 2,6-di­hydroxybenzoic acid is 0.08, 0.15, 0.39 and 0.38, respectively. The site-occupancy values were obtained by free refinement of carbon or oxygen electron density to give the lowest *R* factor.

**Figure 6:**
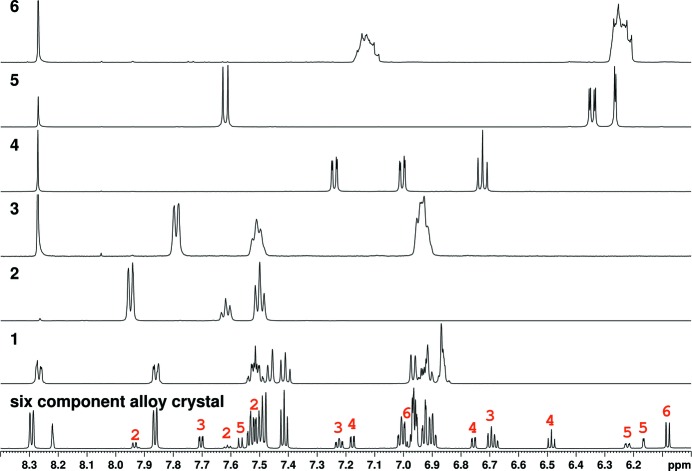
Stacked ^1^H NMR plots of the hexacomponent (123456) salt-alloy crystal along with the five carboxylic acid units (2–6, indicated in black font). The resonances corresponding to the individual carb­oxy­lic acid components in the spectrum of the hexanary crystal are indicated in red.

The crystal packing diagrams of pentanary, quaternary and ternary alloys (Fig. 5 ▸) are similar except for the difference in the hy­droxy-substituted benzoic acid portion of the structure. The similar packing of 2- to 6-component organic alloys is driven by the strong ionic 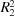
(9) synthon geometry, which is a recurring motif in all of the crystal structures. The remaining alkyl and phenyl groups adjust with the close packing of molecules. DSC of the 6-component organic-salt alloy showed a single, sharp endotherm at 173°C which is neither the melting point of naftopidil, nor matches with any of the single-component acids, nor the binary salts of naftopidil with acids (Figs. 4 ▸ *c* and S5*a*). Further characterization of the hexanary alloy (123456) was carried out using NMR spectroscopy, large single crystals of the alloy were dissolved in DMSO-*d*_6_ and ^1^H, ^13^C, COSY, TOCSY, HSQC and HMBC were acquired along with the individual ^1^H NMR of all six constituent components (Fig. 6 ▸). A correlative analysis of these spectra revealed the presence of all the six individual components in the salt-alloy crystal. The integral ratios of ^1^H NMR resonances of the crystal support the presence of (2) ∼8%, (3) ∼29%, (4) ∼24%, (5) ∼17% and (6) ∼22% and naftopidil (1) 100%. The occupancy values from the SOF of acids in the crystal structure are (2) 0.12, (3) 0.27, (4) 0.14, (5) 0.20 and (6) 0.27. The maximum discrepancy between the NMR and single-crystal occupancy values (8–10%) could be the result of natural variation in the stoichiometry of the components in the hexanary alloy crystals and/or slight inhomogeneity of the NMR sample not being a single crystal. The bulk purity was established by PXRD (Fig. 4 ▸ *d*). A series of ^1^H NMR experiments confirmed the ternary, quaternary and pentanary organic alloys (Figs. S11–S19). The single crystal X-ray structure (Figs. S20–S44), PXRD (Figs. S6*a*–S10), DSC (Figs. S1–S5*b*) and liquid chromatography–mass spectrometry (Figs. S45–S47) of naftopidil salt/alloys are given in the supporting information. In several cases, single-crystal structures were harvested and the structures were satisfactorily solved by X-ray diffraction. For others, crystals were obtained but the quality was not satisfactory for X-ray data collection on a laboratory diffractometer and so complementary NMR was used. The controlled synthesis of multicomponent crystals has applications in the pharmaceutical industry for the formulation of multicomponent drugs as cocrystals and solid solutions. In certain drugs the side effects can be minimized, while administration of two different drugs with synergistic action could mitigate side effects (Zhao *et al.*, 2013 ▸; Prost *et al.*, 2015 ▸). The efficacy of the 1:1 cocrystal-salt combination is enhanced in Valsartan (angiotensin receptor blocker) and Sacubitril (neprilsin inhibitor), Entresto (LCZ696 clinical candidate) (Jessup, 2014 ▸; McMurray *et al.*, 2014 ▸), with a reduced risk of cardiovascular death during hospitalization of patients. Diverse applications of cocrystals and solid solutions and eutectics (multicomponent crystals) have been reported (Goldberg *et al.*, 1966 ▸; Cherukuvada & Nangia, 2014 ▸; Braga *et al.*, 2009 ▸; Romasanta *et al.*, 2017 ▸; Mishra *et al.*, 2015 ▸, Lusi, 2018 ▸).

## 4. Conclusions   

We have successfully designed a hexacomponent organic-salt alloy of an active pharmaceutical ingredient by geometrical and chemical optimization based on a strong ionic 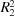
(9) synthon. The generality of our method is demonstrated by the synthesis of over 26 organic-salt alloys by considering five isostructural binary salts which could lead to the manufacture of multicomponent pharmaceutical adducts as improved drugs.

## Supplementary Material

Crystal structure: contains datablock(s) 12, 13, 14, 15, 16, 123, 124, 126, 134, 135, 136, 145, 146, 1234, 1235, 1236, 1245, 1246, 1346, 1356, 1456, 12345, 12346, 12356, 123456, platon_pl. DOI: 10.1107/S2052252518014057/ed5016sup1.cif

Supporting tables, figures and schemes. DOI: 10.1107/S2052252518014057/ed5016sup2.pdf

CCDC references: 1817125, 1817126, 1817127, 1817128, 1817129, 1817130, 1817146, 1817147, 1817148, 1817149, 1817150, 1817151, 1817158, 1817159, 1817160, 1817161, 1817163, 1817164, 1817165, 1817166, 1817167, 1817168, 1817169, 1817170, 1840048, 1874903

## Figures and Tables

**Table 1:** Structural data for the binary salts of naftopidil

Binary salt	Space group	*a* (Å)	*b* (Å)	*c* (Å)	β (°)	*V* (Å^3^)
(12)	*P*2_1_/*c*	11.0695 (2)	17.7360 (3)	13.5179 (3)	97.3794 (19)	2631.97
(13)	*P*2_1_/*c*	11.2350 (5)	17.6516 (6)	13.4798 (6)	96.269 (4)	2657.27
(14)	*P*2_1_/*c*	11.3258 (4)	17.8349 (7)	13.4077 (7)	95.800 (4)	2694.42
(15)	*P*2_1_/*c*	10.843 (4)	17.862 (6)	13.913 (6)	91.715 (12)	2693.43
(16)	*P*2_1_	11.4733 (3)	17.6286 (5)	13.6530 (4)	93.290 (2)	2756.88

**Table 2:** Salt and salt-alloy synthesis: naftopidil (1) with five aromatic acids Crystallographic information for the organic salts and salt alloys is given in Table S2.

Naftopidil	Coformer	Binary†	Hexanary‡	Pentanary§	Quaternary¶	Ternary††	
(1)	(2)	(12)	(123456)	(12356)‡‡	(1234)	(123)‡‡	
	(3)	(13)		(12346)‡‡	(1235)	(124)	
		(14)		(12345)‡‡	(1236)	(125)§§	
	(4)	(15)		(13456)‡‡ §§	(1245)	(126)	
		(16)		(12456)‡‡ §§	(1246)	(134)	
	(5)				(1256)§§	(135)	
					(1345)§§	(136)	
	(6)				(1346)	(145)	
					(1356)	(146)	
					(1456)‡‡	(156)§§	

† Binary salt: 1 mmol of (1) and 1 mmol of each acid.

‡ Hexacomponent (123456) alloy: 1 mmol of (1) and 0.2 mmol of each acid.

§ Pentanary alloy: 1 mmol of (1) and 0.25 mmol of each aromatic acid.

¶ Quaternary alloy: 1 mmol of (1) and 0.33 mmol of each acid.

†† Ternary alloy: 1 mmol of (1) and 0.50 mmol of each acid.

‡‡ Stacked ^1^H NMR plots for these crystals along with their components are provided in the supporting information.

§§ Crystalline solids were obtained, but the single-crystal data quality was poor.
