# Supplementary material for: Systematic synthesis of a 6-component organic-salt alloy of naftopidil, and pentanary, quaternary and ternary multicomponent crystals
Source: IUCrJ. 2018 Oct 24;5(Pt 6):816–22. doi: 10.1107/S2052252518014057 (PMC6211519; doi:10.1107/S2052252518014057)
Supplement: Supplementary file 2 [file m-05-00816-sup2.pdf]

IUCrJ

Volume 6 (2018)

Supporting information for article:

Systematic synthesis of a 6-component organic-salt alloy of naftopidil, and pentanary, quaternary and ternary multicomponent crystals

Rambabu Dandela, Srinu Tothadi, Udaya Kiran Marelli and Ashwini Nangia

Systematic Synthesis of Six Component Organic Salt Alloy of Naftopidil and Pentanary, Quaternary and Ternary Multicomponent Crystals

Rambabu Dandela,[a*] Srinu Tothadi,[a] Udaya Kiran Marelli,[ab] and Ashwini Nangia[ac*]

[a] Organic Chemistry Division, CSIR-National Chemical Laboratory, Dr. Homi Bhabha Road, Pune, Maharashtra, 411 008, India

[b] Central NMR Facility, CSIR-National Chemical Laboratory, Dr. Homi Bhabha Road, Pune, Maharashtra, 411 008, India

[c] School of Chemistry, University of Hyderabad, Prof. C. R. Rao Road, Gachibowli, Hyderabad, Telangana, 500 046, India

Correspondence email: r.dandela@ncl.res.in; ashwini.nangia@gmail.com; ak.nangia@ncl.res.in

Rambabu Dandela and Srinu Tothadi equally contributed to the work

Table S1 Cocrystallization of naftopidil with five aromatic acids.

Solid form	Naftopidil (mmol)	Coformer (mg, mmol)	Method, Solvent	Solvent of crystallization
Hexacomponent (123456) alloy	1.0 mmol	Each coformer 0.2 mmol	Mortar-pestle grinding, CH_3OH (2-3 drops)	CH_3OH (30-40 mg of crystalline material was taken in 5 mL)
Pentanary (13456) alloy<br>Pentanary (12456) alloy<br>Pentanary (12356) alloy<br>Pentanary (12346) alloy Pentanary (12345) alloy	1.0 mmol	Each coformer 0.25 mmol	Mortar-pestle grinding, CH_3OH (2-3 drops)	CH_3OH (30-40 mg of crystalline material was taken in 5 mL)
Quaternary (1234) alloy<br>Quaternary (1235) alloy<br>Quaternary (1236) alloy<br>Quaternary (1245) alloy<br>Quaternary (1246) alloy<br>Quaternary (1256) alloy<br>Quaternary (1345) alloy<br>Quaternary (1346) alloy<br>Quaternary (1356) alloy<br>Quaternary (1456) alloy	1.0 mmol	Each coformer 0.333 mmol	Mortar-pestle grinding, CH_3OH (2-3 drops)	CH_3OH (30-40 mg of crystalline material was taken in 5 mL)
Ternary (123) alloy<br>Ternary (124) alloy<br>Ternary (125) alloy<br>Ternary (126) alloy<br>Ternary(134) alloy<br>Ternary (135) alloy<br>Ternary (136) alloy<br>Ternary (145) alloy<br>Ternary (146) alloy	1.0 mmol	Each coformer 0.5 mmol	Mortar-pestle grinding, CH_3OH (2-3 drops)	(30-40 mg of crystalline material was taken in 5 mL)

Ternary (156) alloy				
Binary salts (12)<br>Binary salts (13)<br>Binary salts (14)<br>Binary salts (15)<br>Binary salts (16)	1.0 mmol	Each coformer 1.0 mmol	Mortar-pestle grinding, CH_3OH (2-3 drops)	(30-40 mg of crystalline material was taken in 5 mL)
Naftopidil-2,4,6-trihydroxybenzoic acid salt	1.0 mmol	1.0 mmol	Mortar-pestle grinding, CH_3OH (2-3 drops)	(30-40 mg of crystalline material was taken in 5 mL)

Table S2 Crystallographic data of salts and salt alloys of Naftopidil (1).

	12	13	14	15	16
Empirical Formula	$C_{24}H_{29}N_2O_3\cdot C_7H_5O_2$	$C_{24}H_{29}N_2O_3\cdot C_7H_5O_3$	$C_{24}H_{29}N_2O_3\cdot C_7H_5O_4$	$C_{24}H_{29}N_2O_3\cdot C_7H_5O_4$	$C_{24}H_{29}N_2O_3\cdot C_{24}H_{28}N_2O_3\cdot 2(C_7H_5O_4)$
Formula weight	514.60	530.60	546.60	546.60	1092.19
Crystal System	Monoclinic	Monoclinic	Monoclinic	Monoclinic	Monoclinic
Space Group	$P2_1/c$	$P2_1/c$	$P2_1/c$	$P2_1/c$	$P2_1$
T (K)	293(2)	293(2)	293(2)	100(2)	293(2)
a (Å)	11.0695 (2)	11.2350 (5)	11.3258 (4)	10.843 (4)	11.4733 (3)
b (Å)	17.7360 (3)	17.6516 (6)	17.8349 (7)	17.862 (6)	17.6286 (5)
c (Å)	13.5179 (3)	13.4798 (6)	13.4077 (7)	13.913 (6)	13.6530 (4)
α (°)	90	90	90	90	90
β (°)	97.3794 (19)	96.269 (4)	95.800 (4)	91.715 (12)	93.290 (2)
γ (°)	90	90	90	90	90
V (Å^3)	2632.00 (9)	2657.27 (18)	2694.4 (2)	2693.4 (17)	2756.89 (13)
D_{calc} (g cm^{-3})	1.299	1.326	1.347	1.348	1.316
Z	4	4	4	4	2
F(000)	1096	1128	1160	1160	1158
Residual electron density	0.44, -0.29	0.32, -0.17	0.41, -0.29	0.81, -0.35	0.21, -0.19

$\Delta\rho_{max}$, $\Delta\rho_{min}$ (e Å^{-3})					
h range	-13→ 13	-13 → 13	-13 → 13	-14→ 14	-13→ 11
k range	-21 → 21	-20 → 13	-15 → 21	-24 → 24	-19 → 20
l range	-16 → 16	-14 → 16	-14 → 15	-18 → 18	-14 → 16
measured reflections,	24778	11084	11635	66674	11695
independent reflections,	4626	4667	4734	6923	8293
Reflections with $I > 2\sigma(I)$	3831	3368	3717	5449	6342
R_{int}	0.042	0.040	0.041	0.068	0.049
R_1 [$I > 2\sigma(I)$]	0.057	0.064	0.088	0.054	0.061
wR_2 (all)	0.153	0.170	0.256	0.142	0.170
Goodness of fit	1.13	1.10	1.15	1.07	1.05
X-ray diffractometer	Rigaku OD	Rigaku OD	Rigaku OD	BRUKER APEX	Rigaku OD
CCDC No.	1817128	1817160	1817125	1817161	1817170

Naftopidil ternary alloys: 125 and 156 were characterized by PXRD/NMR and are not listed here.

	123	124	126	134	135
Empirical Formula	$C_7H_5O_{2.9}\cdot C_{24}H_{29}N_2O_3$	$C_7H_{5.42}O_{3.42}\cdot C_{24}H_{29}N_2O_3$	$C_7H_{4.84}O_{2.4}\cdot C_7H_5O_{3.32}\cdot 2(C_{24}H_{29}N_2O_3)$	$C_7H_5O_{3.51}\cdot C_{24}H_{29}N_2O_3$	$C_7H_{5.55}O_{3.14}\cdot C_{24}H_{29}N_2O_3$
Formula weight	529.00	537.74	1056.56	538.76	533.39
Crystal System	Monoclinic	Monoclinic	Monoclinic	Monoclinic	Monoclinic
Space	$P2_1/c$	$P2_1/c$	$P2_1$	$P2_1/c$	$P2_1/c$

Group					
T (K)	100(2)	100(2)	100(2)	100(2)	100(2)
a (Å)	11.197 (2)	11.282 (4)	11.346 (2)	11.253 (4)	11.176 (4)
b (Å)	17.627 (4)	17.856 (7)	17.508 (4)	17.660 (6)	17.608 (5)
c (Å)	13.199 (3)	13.460 (5)	13.400 (3)	13.247 (4)	13.305 (4)
α (°)	90	90	90	90	90
β (°)	96.347 (6)	96.060 (12)	98.15 (3)	95.787 (11)	95.426 (10)
γ (°)	90	90	90	90	90
V (Å^3)	2589.0 (9)	2696.3 (17)	2635.0 (10)	2619.1 (15)	2606.6 (14)
D_{calc}(g cm^{-3})	1.357	1.325	1.332	1.366	1.359
Z	4	4	2	4	4
F(000)	1125	1143	1123	1144	1135
Residual electron density $\Delta\rho_{max}$, $\Delta\rho_{min}$ (e Å^{-3})	0.44, -0.36	0.32, -0.33	0.29, -0.25	0.73, -0.36	0.35, -0.65
h range	-15→ 15	-15 → 15	-16 → 16	-17→ 16	-15→ 14
k range	-24 → 24	-23 → 23	-24 → 24	-27 → 27	-24 → 25
l range	-18 → 18	-17 → 17	-19 → 19	-18 → 20	-17 → 18
measured reflections,	53638	88550	65036	57701	39100
independent reflections,	7114	6635	15959	9884	7888
Reflections with $I > 2\sigma(I)$	6706	5411	10798	8381	6366
R_{int}	0.025	0.056	0.071	0.039	0.047
R_1 [$I > 2\sigma(I)$]	0.041	0.070	0.083	0.052	0.062

]					
wR_2 (all)	0.122	0.231	0.164	0.154	0.140
Goodness of fit	1.10	1.64	1.06	1.10	1.12
X-ray diffractometer	BRUKER APEX	Rigaku OD	BRUKER APEX	BRUKER APEX	BRUKER APEX
CCDC No.	1817165	1817164	1817158	1817169	1817149

Naftopidil ternary alloys

	136	145	146
Empirical Formula	$C_7H_5O_{3.47}{\cdot}C_{24}H_{29}N_2O_3$	$C_7H_5O_4{\cdot}C_{24}H_{29}N_2O_3$	$C_7H_4O_4{\cdot}C_{24}H_{29}N_2O_3$
Formula weight	538.12	546.60	545.59
Crystal System	Monoclinic	Monoclinic	Monoclinic
Space Group	$P2_1/c$	$P2_1/c$	$P2_1/c$
T (K)	100(2)	100(2)	100(2)
a (Å)	11.487 (7)	11.195 (15)	11.475 (6)
b (Å)	17.514 (10)	17.69 (3)	17.669 (9)
c (Å)	13.310 (9)	13.449 (18)	13.248 (7)
α (°)	90	90	90
β (°)	95.95 (3)	94.40 (5)	94.87 (2)
γ (°)	90	90	90
V (Å^3)	2663 (3)	2655 (7)	2676 (2)
D_{calc}(g cm^{-3})	1.342	1.367	1.354
Z	4	4	4
F(000)	1143	1160	1156
Residual electron	0.25, -0.34	0.51, -0.38	0.58, -0.49

density $\Delta\rho_{max}$, $\Delta\rho_{min}$ (e Å^{-3})			
h range	-15→ 15	-15 → 15	-16 → 16
k range	-23 → 23	-25 → 25	-25 → 25
l range	-17 → 17	-19 → 19	-18 →18
measured reflections,	76404	63575	88884
independent reflections,	6567	8027	8099
Reflections with $I > 2\sigma(I)$	4598	6474	6717
R_{int}	0.166	0.060	0.073
R_1 [$I > 2\sigma(I)$]	0.104	0.099	0.111
wR_2 (all)	0.222	0.219	0.251
Goodness of fit	1.12	1.18	1.21
X-ray diffractometer	BRUKER APEX	BRUKER APEX	BRUKER APEX
CCDC No.	1817150	1817129	1817146

Naftopidil quaternary alloy: 1256 and 1345 were characterized by PXRD/NMR and are not listed here.

	1234	1235	1236	1245
Empirical Formula	$C_7H_{5.12}O_{3.33}\cdot C_{24}H_{29}N_2O_3$	$C_7H_5O_{3.17}\cdot C_{24}H_{29}N_2O_3$	$C_7H_5O_{3.27}\cdot C_{24}H_{29}N_2O_3$	$C_7H_5O_{3.46}\cdot C_{24}H_{29}N_2O_3$
Formula weight	536.03	533.35	534.95	537.99
Crystal System	Monoclinic	Monoclinic	Monoclinic	Monoclinic
Space Group	$P2_1/c$	$P2_1/c$	$P2_1/c$	$P2_1/c$
T (K)	297(2)	293(2)	293(2)	100(2)

a (Å)	11.296 (2)	11.20 (2)	11.369 (4)	11.102 (5)
b (Å)	17.807 (4)	17.71 (3)	17.660 (6)	17.701 (9)
c (Å)	13.459 (3)	13.40 (3)	13.539 (5)	13.437 (6)
α (°)	90	90	90	90
β (°)	96.00 (3)	94.85 (7)	95.632 (17)	94.667 (18)
γ (°)	90	90	90	90
V (Å^3)	2692.6 (10)	2648 (9)	2705.1 (16)	2632 (2)
D_{calc}(g cm^{-3})	1.322	1.338	1.313	1.358
Z	4	4	4	4
F(000)	1139	1134	1137	1143
Residual electron density $\Delta\rho_{max}$, $\Delta\rho_{min}$ (e Å^{-3})	0.25, -0.30	0.68, -0.72	0.30, -0.25	0.56, -0.61
h range	-14→ 14	-15 → 15	-16 → 16	-14→ 14
k range	-22→ 22	-24 → 25	-25→ 24	-23 → 23
l range	-16 → 16	-19 → 19	-19 → 19	-17 → 17
measured reflections,	62700	67030	90604	61552
independent reflections,	5404	7947	8175	6497
Reflections with $I > 2\sigma(I)$	4670	5620	5373	5857
R_{int}	0.039	0.142	0.050	0.048
R_1 [$I > 2\sigma(I)$]	0.045	0.106	0.089	0.099
wR_2 (all)	0.139	0.208	0.192	0.256
Goodness of fit	1.08	1.08	1.07	1.00
X-ray diffractometer	BRUKER APEX	BRUKER APEX	BRUKER APEX	BRUKER APEX

CCDC No.	1817159	1817130	1817147	1817148

Naftopidil quaternary alloy:

	1246	1346	1356	1456
Empirical Formula	$C_7H_5O_{3.4}\cdot C_{24}H_{29}N_2O_3$	$C_7H_5O_{3.81}\cdot C_{24}H_{29}N_2O_3$	$C_7H_{4.91}O_{3.51}\cdot C_7H_6O_{3.52}\cdot 2(C_{24}H_{29}N_2O_3)$	$C_7H_5O_4\cdot C_{24}H_{29}N_2O_3$
Formula weight	537.03	543.56	1078.60	546.60
Crystal System	Monoclinic	Monoclinic	Monoclinic	Monoclinic
Space Group	$P2_1/c$	$P2_1/c$	$P2_1$	$P2_1/c$
T (K)	293(2)	293(2)	100(2)	100(2)
a (Å)	11.410 (4)	11.405	11.246 (10)	11.319 (2)
b (Å)	17.772 (6)	17.746	17.607 (16)	17.672 (4)
c (Å)	13.550 (5)	13.511	13.523 (13)	13.391 (3)
α (°)	90	90	90	90
β (°)	95.891 (19)	95.49	94.19 (4)	93.90 (3)
γ (°)	90	90	90	90
V ($Å^3$)	2733.1 (17)	2722.0	2670 (4)	2672.4 (10)
D_{calc}(g cm^{-3})	1.305	1.326	1.341	1.359
Z	4	4	2	4
F(000)	1141	1154	1123	1160
Residual electron density $\Delta\rho_{max}$, $\Delta\rho_{min}$ (e $Å^{-3}$)	0.26, -0.21	0.28, -0.32	0.54, -0.29	0.28, -0.33
h range	-14→ 14	-16 → 16	-16 → 16	-13→ 14
k range	-21 → 22	-25 → 25	-25 → 25	-22 → 22
l range	-16 → 16	-19 → 19	-18 → 19	-16 → 16
measured	46089	69144	63374	48278

reflections,				
independent reflections,	5487	8182	16100	5387
Reflections with $I > 2\sigma(I)$	4300	5170	8744	4822
R_{int}	0.056	0.057	0.112	0.040
R_1 $[I > 2\sigma(I)]$	0.075	0.081	0.097	0.072
wR_2 (all)	0.165	0.225	0.198	0.154
Goodness of fit	1.07	1.10	1.04	1.19
X-ray diffractometer	BRUKER APEX	BRUKER APEX	BRUKER APEX	BRUKER APEX
CCDC No.	1817151	1817162	1817168	1817167

Naftopidil pentanary alloy: 13456 and 12456 were characterized by PXRD/NMR and are not listed here.

	12345	12346	12356
Empirical Formula	$C_{7.1}H_{5.33}O_{3.35}{\cdot}C_{24}H_{29}N_2O_3$	$C_7H_5O_{3.69}{\cdot}C_{24}H_{29}N_2O_3$	$C_7H_{4.8}O_{3.31}{\cdot}C_7H_5O_{3.32}{\cdot}2(C_{24}H_{29}N_2O_3)$
Formula weight	537.73	541.66	1071.08
Crystal System	Monoclinic	Monoclinic	Monoclinic
Space Group	$P2_1/c$	$P2_1/c$	$P2_1$
T (K)	100(2)	100(2)	100(2)
a (Å)	11.153 (4)	11.405 (5)	11.237 (5)
b (Å)	17.701 (5)	17.591 (8)	17.571 (6)
c (Å)	13.336 (4)	13.269 (6)	13.374 (6)
α (°)	90	90	90
β (°)	95.232 (17)	95.526 (15)	94.584 (16)

γ (°)	90	90	90
V (Å^3)	2621.8 (13)	2650 (2)	2632.4 (18)
D_{calc}(g cm^{-3})	1.362	1.358	1.351
Z	4	4	2
F(000)	1143	1150	1138
Residual electron density $\Delta\rho_{max}$, $\Delta\rho_{min}$ (e Å^{-3})	0.42, -0.50	0.47, -0.49	0.52, -0.48
h range	-15→ 15	-16 → 16	-15 → 15
k range	-25→ 25	-25 → 25	-24→ 24
l range	-18 → 18	-16 → 18	-18 → 18
measured reflections,	63397	86570	80672
independent reflections,	7929	8037	14648
Reflections with I> 2σ(I)	6429	6291	11616
R_{int}	0.055	0.064	0.046
R_1 [I >2σ(I)]	0.064	0.084	0.053
wR_2 (all)	0.173	0.178	0.153
Goodness of fit	1.17	1.07	1.07
X-ray diffractometer	BRUKER APEX	BRUKER APEX	BRUKER APEX
CCDC No.	1817166	1817126	1817127

Naftopidil hexanary alloy:

	123456

Empirical Formula	$C_7H_5O_{3.49}\cdot C_{24}H_{29}N_2O_3$
Formula weight	538.44
Crystal System	Monoclinic
Space Group	$P2_1/c$
T (K)	100(2)
a (Å)	11.279 (5)
b (Å)	17.633 (7)
c (Å)	13.377 (6)
α (°)	90
β (°)	94.51 (2)
γ (°)	90
V (Å^3)	2652.2 (18)
D_{calc}(g cm^{-3})	1.348
Z	4
F(000)	1144
Residual electron density $\Delta\rho_{max}$, $\Delta\rho_{min}$ (e Å^{-3})	0.28, -0.24
h range	-14→ 14
k range	-21→ 21
l range	-16 → 16
measured reflections,	31190
independent reflections,	5299
Reflections with $I > 2\sigma(I)$	4387
R_{int}	0.054
R_1 [$I > 2\sigma(I)$]	0.072
wR_2 (all)	0.162

Goodness of fit	1.19
X-ray diffractometer	BRUKER APEX
CCDC No.	1817163

Naftopidil-2,4,6-trihydroxybenzoic acid salt:

	Naftopidil-2,4,6-trihydroxybenzoic acid salt
Empirical Formula	$C_7H_5O_5 \cdot C_{24}H_{29}N_2O_3$
Formula weight	562.60
Crystal System	Monoclinic
Space Group	$P2_1/c$
T (K)	100(2)
a (Å)	11.074 (2)
b (Å)	17.895 (3)
c (Å)	14.000 (2)
α (°)	90
β (°)	92.85 (8)
γ (°)	90
V (Å^3)	2770.9 (8)
D_{calc}(g cm^{-3})	1.349
Z	4
F(000)	1192
Residual electron density $\Delta\rho_{max}$, $\Delta\rho_{min}$ (e Å^{-3})	0.41, -0.48
h range	-15→ 15
k range	-25→ 25

l range	-19 → 19
measured reflections,	14029
independent reflections,	8405
Reflections with *I*> 2σ(*I*)	6782
R_{int}	0.066
R_1 [I >2σ(I)]	0.046
wR_2 (all)	0.142
Goodness of fit	1.11
X-ray diffractometer	BRUKER APEX
CCDC No.	1840048

Table S3 s.o.f. derived from X-ray crystal structure refinement.

		**1**	**2**	**3**	**4**	**5**	**6**
1	Hexacomponent (123456) alloy	1.00	0.12	0.27	0.27	0.14	0.20
2	Pentanary (12346) alloy	1.00	0.8	0.15	0.39		0.38
3	Pentanary (12345) alloy	1.00	0.23	0.19	0.35	0.23	
4	Pentanary (12356) alloy	1.00	0.095	0.495		0.20	0.21
5	Quaternary (1234) alloy	1.00	0.12	0.43	0.45		
6	Quaternary (1236) alloy	1.00	0.10	0.53			0.37
7	Quaternary (1246) alloy	1.00	0.16		0.42		0.42
8	Quaternary (1346) alloy	1.00		0.19	0.43		0.38
9	Quaternary (1456) alloy	1.00			0.46	0.20	0.34
10	Quaternary (1235) alloy	1.00	0.13	0.57		0.30	
11	Quaternary (1245) alloy	1.00	0.27		0.37	0.36	
12	Quaternary 1356 alloy	1.00		0.485		0.26	0.255
13	Ternary (123) alloy	1.00	0.10	0.90			
14	Ternary (124) alloy	1.00	0.29		0.71		
15	Ternary (126) alloy	1.00	0.57				0.43
16	Ternary (134) alloy	1.00		0.49	0.51		

17	Ternary (135) alloy	1.00		0.86		0.14	
18	Ternary (136) alloy	1.00		0.53			0.47
19	Ternary (145) alloy	1.00		0.65	0.35		
20	Ternary (146) alloy	1.00			0.53		0.47

Table S4 Refcodes of R_2^2 (9) motif.

CSD Refocodes containing the O–H⋯O + N–H⋯O synthon

**Refcodes**	**Refcodes**	**Refcodes**	**Refcodes**
LEBSIX	HMGLUT	OJUQER	VABSAU
TEGJOH	HUZQIE	ORUWUU	VIMNOL10
YEDCUI	ICIMOX	ORUXAB	WANKIF
ASAPIV	IHUFOG	PCACAP	WANKOL
AXOVIU	INEDOV	PEPFIA	WAXZAY
AYEPIF	IPUMUC	PETQAH	WAXZEC
AZABEI	IVULOB	PUKRET	WELYUH
BALXER	JAPWIJ	QAKQIE	WIRREU
BURXIX	JUHLUT10	QAWSUC	WIRRIY
CABPOK	JUHLUT12	QORROE	WIRROE
CAGXEN	KAPZOQ	RONSAQ	WOHRUH
CAZGOA	KAQBAF	RUVNEC	XUHZAD
CIJMEO	KASWEI	SARBUI	XUZBUQ01
CUDCAG	KASXAF	SEMWAI	YAWLIU
CUGPIF	KASXIN	SEQGAX01	YENGOP
CUYBIJ	KEMXIJ	SERGLY	YENGOP01
DETHIU	KEZFIG	SEZLOZ	YIKLUB
DHPROL10	KEZFOM	SOQQUK	YIVCIQ
DINYEE10	KIXBID	SUWBER	YUCSOE
DLSERN02	KIYHOP	TEYSIB	YUFMAN
DOTRAF	KULMOU	TEYSOH	ZASKIQ
DUMJUS	KUZVOR	TEYSUN	ZAVMER
ETOTIS	LEFHIN	TYRXEA10	ZIXTEF
FOVMUZ	LEKRIC	UBUCIG	ZUTRIR
GADXUH	LEWQOV	UDUWEX	ZUYJOU
GADYUI	LEZMIM	UGEYUA	
GEPNEW	LIGZUX	ULICAV01	
GICHIK	LIHBAG	UMIYUM	
GICHOQ	LIHBIO	UPUVOR	
GIDVEU	LIHGAL	UPUVUX	
GISKAW	LSERMH10	UPUWAE	
HEVJAT	MAKXED	UPUWEI	
HEZXAM	MICLAM	URAWER	

HISMAZ	NAJFIO	UREWAQ	
HIVYER	NIDSOK	USOCIP	

CSD version 5.38 updates (Nov 2016). Filters: 3D coordinates determined Only Organics
Number of Hits: 130 Hits shows R_2^2 (9) motif in CSD. Duplicate occurrences of the same motif in the same crystal structures (Refcodes) were removed manually and best unique structure is reported.

Following crystal structure of salts show R_2^2 (9) motif and their CSD refcodes

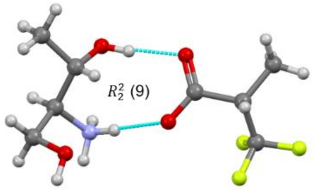

ASAPIV

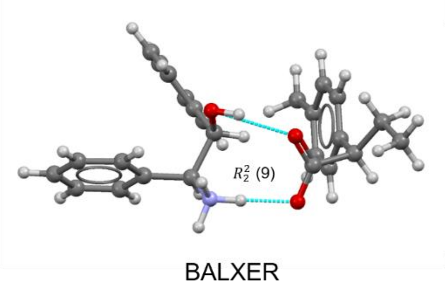

BALXER

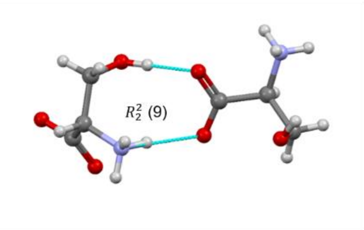

DLSERN02

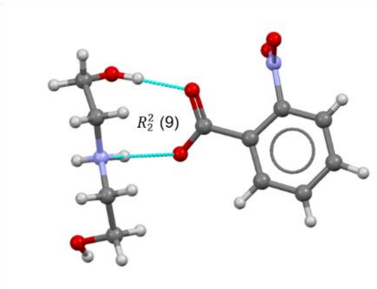

ETOTIS

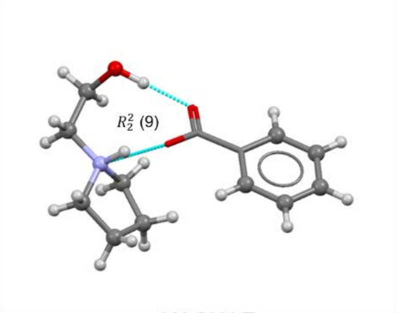

KASXAF

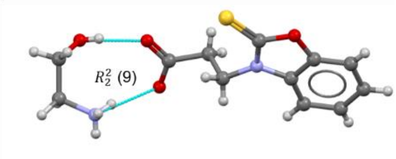

YEDCUI

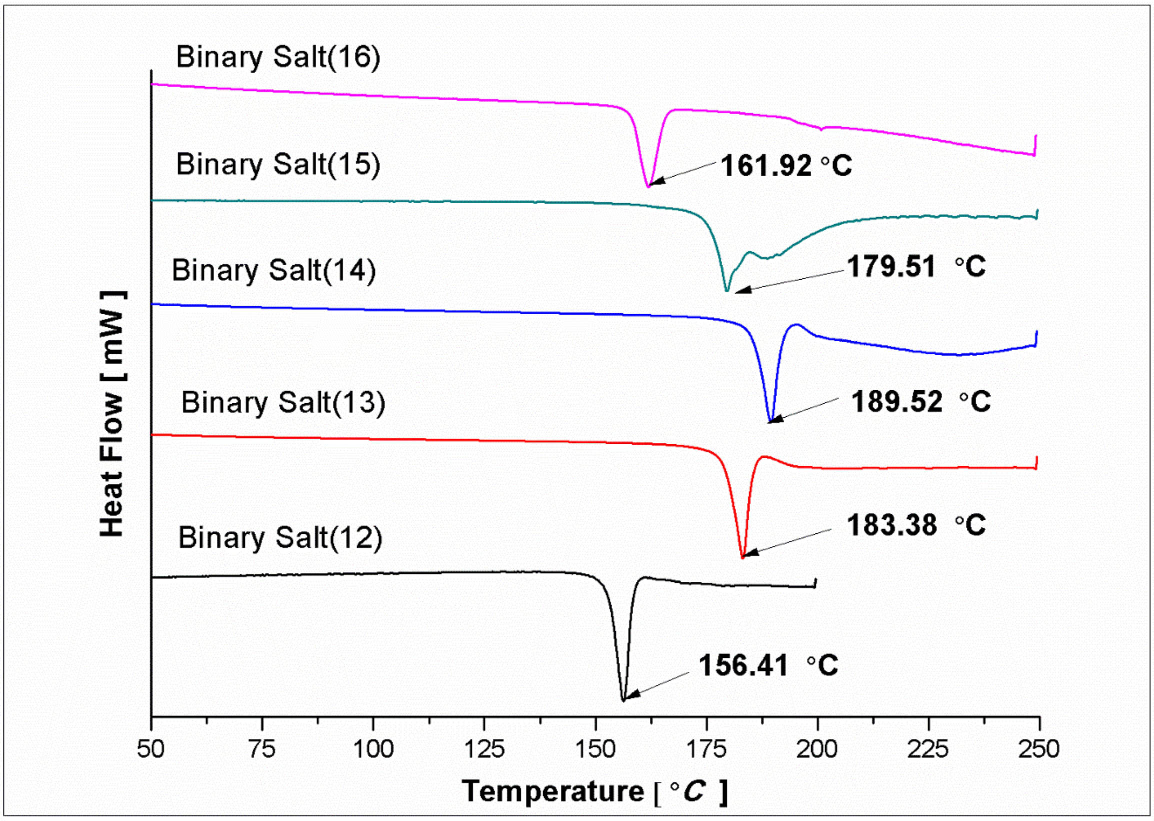

Figure S1 DSC thermogram of binary salts.

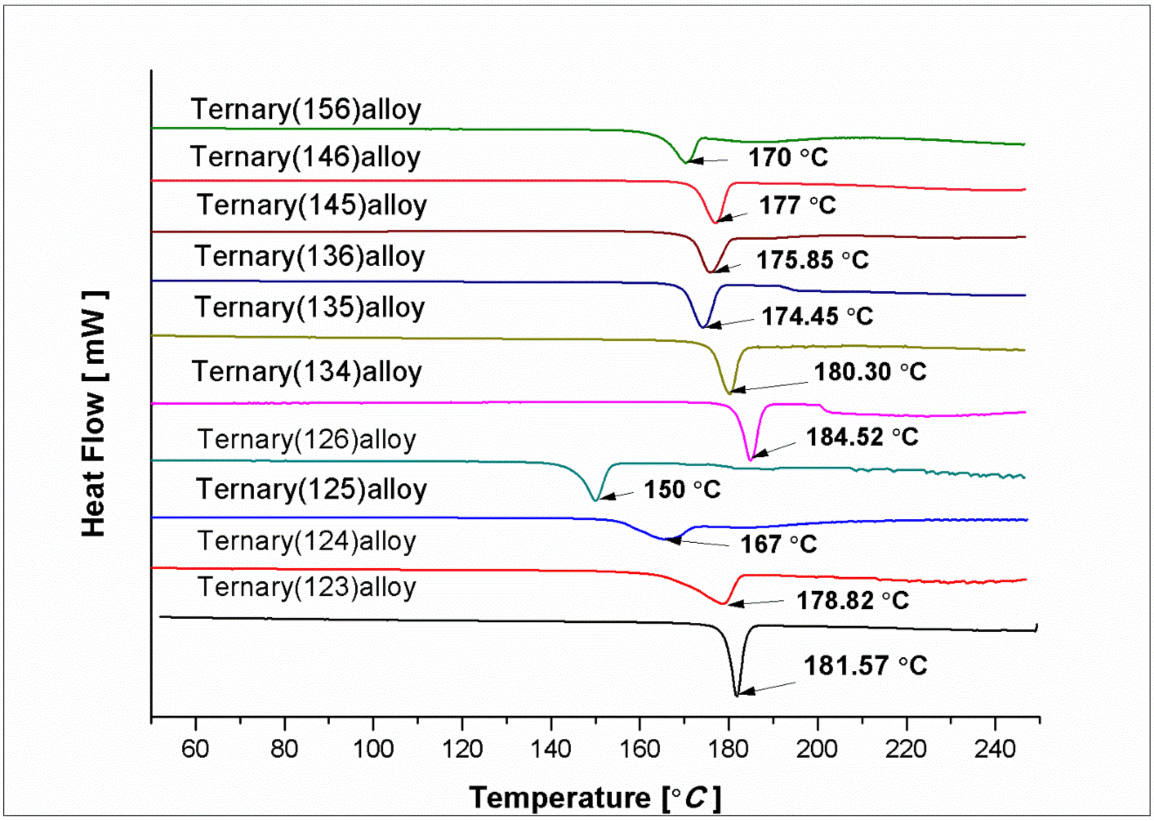

Figure S2 DSC thermogram of ternary alloy.

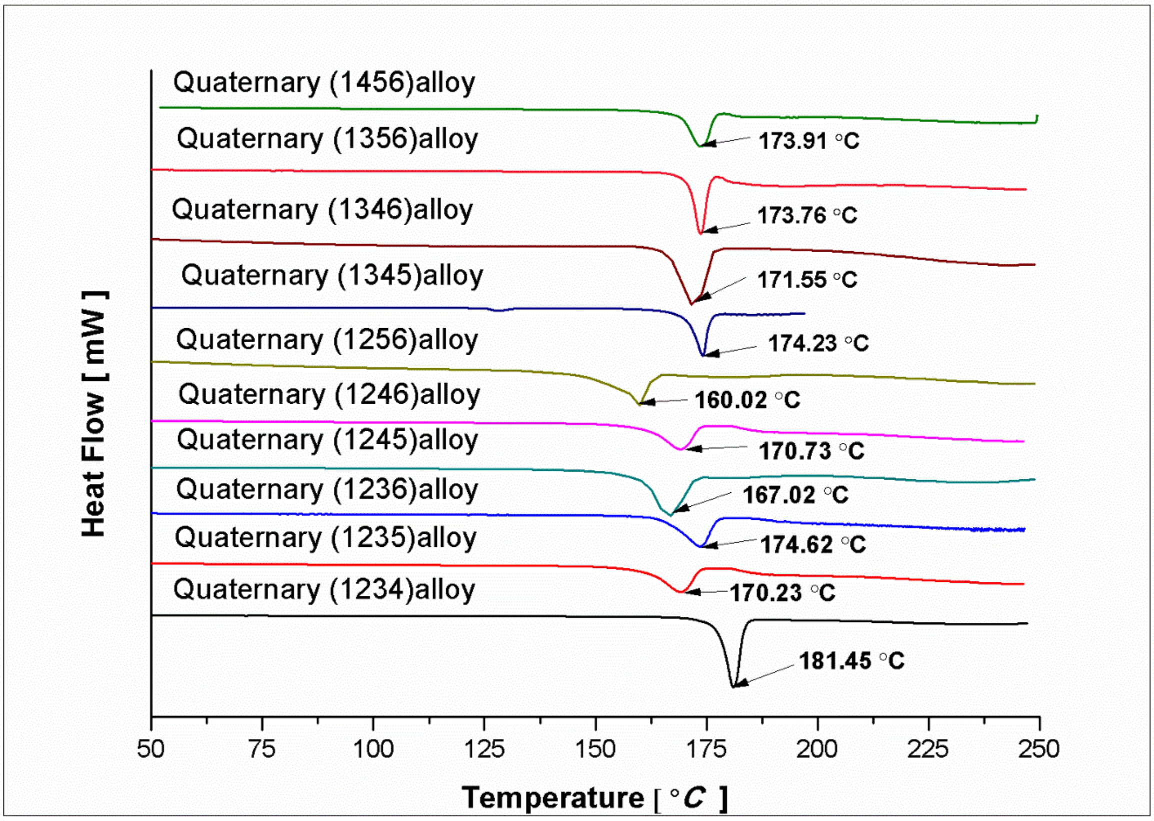

Figure S3 DSC thermogram of quaternary alloy.

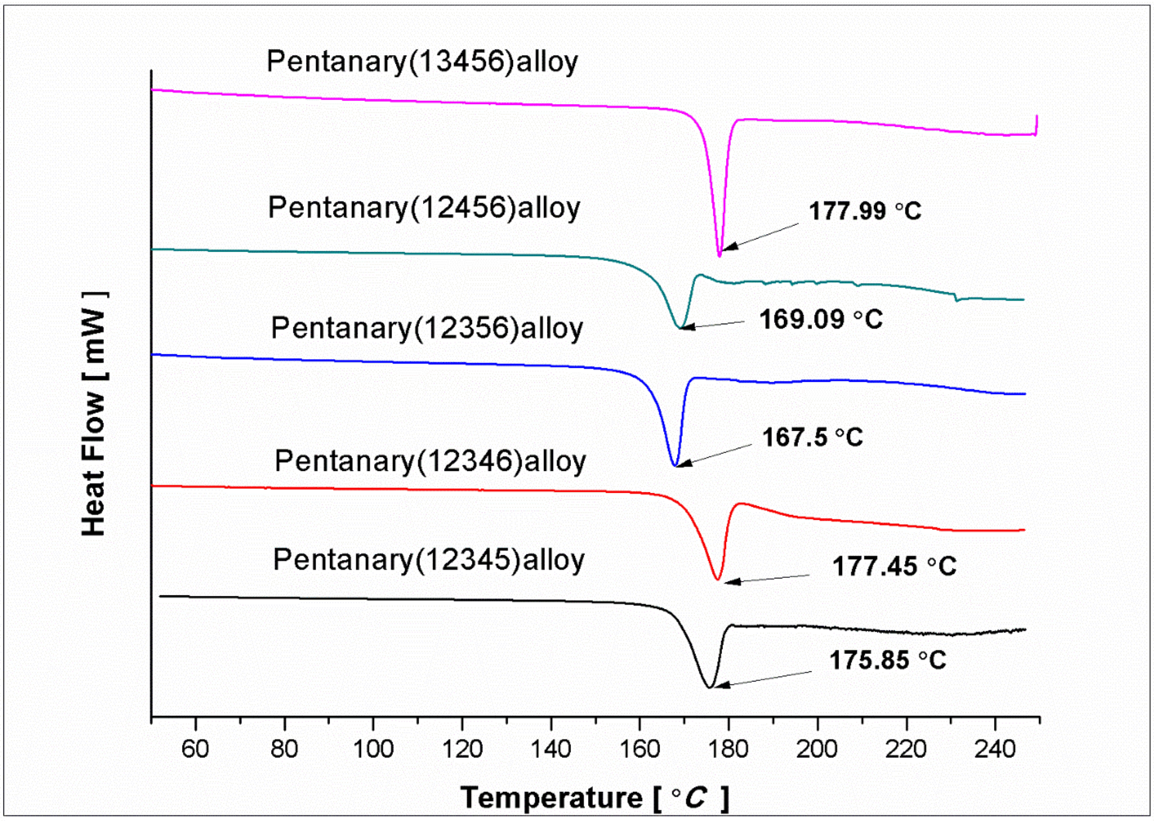

Figure S4 DSC thermogram of Pentanary alloy.

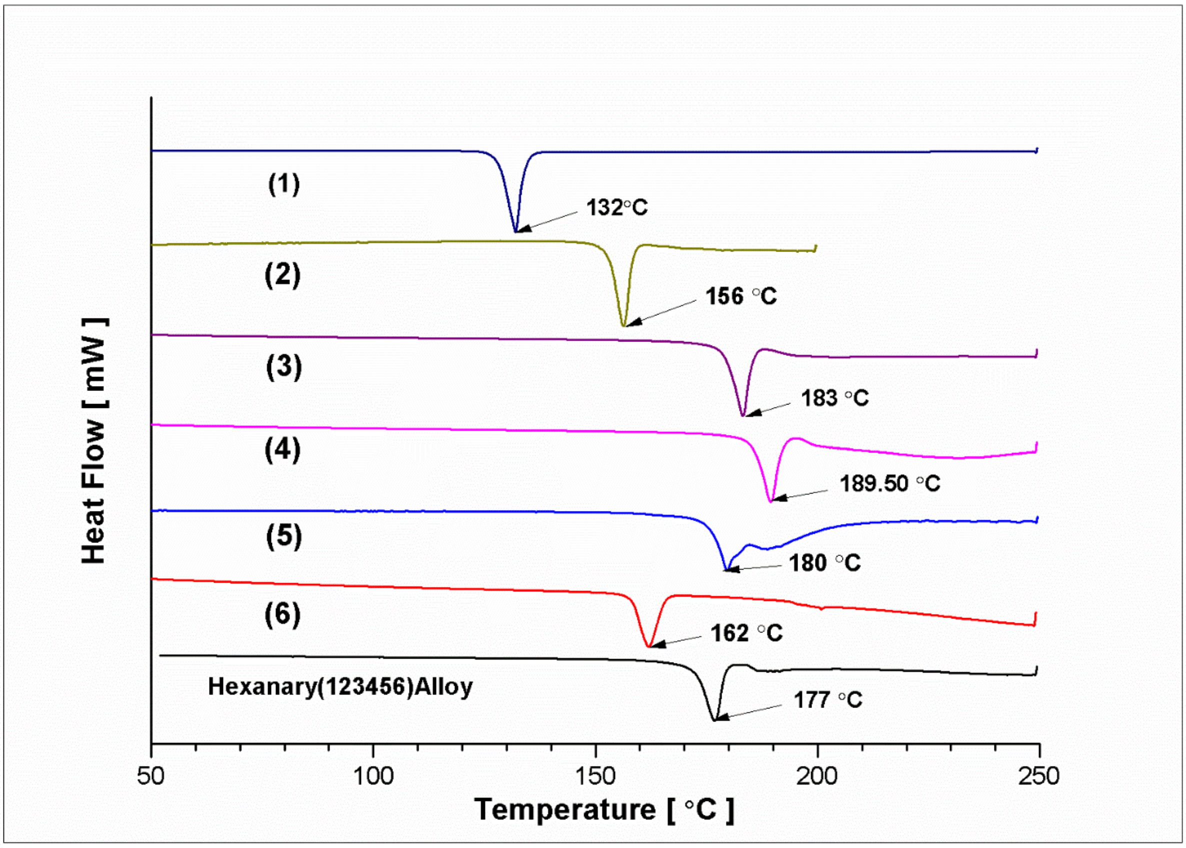

Figure S5a DSC thermogram of hexanary alloy is compared with coformers (aromatic acids).

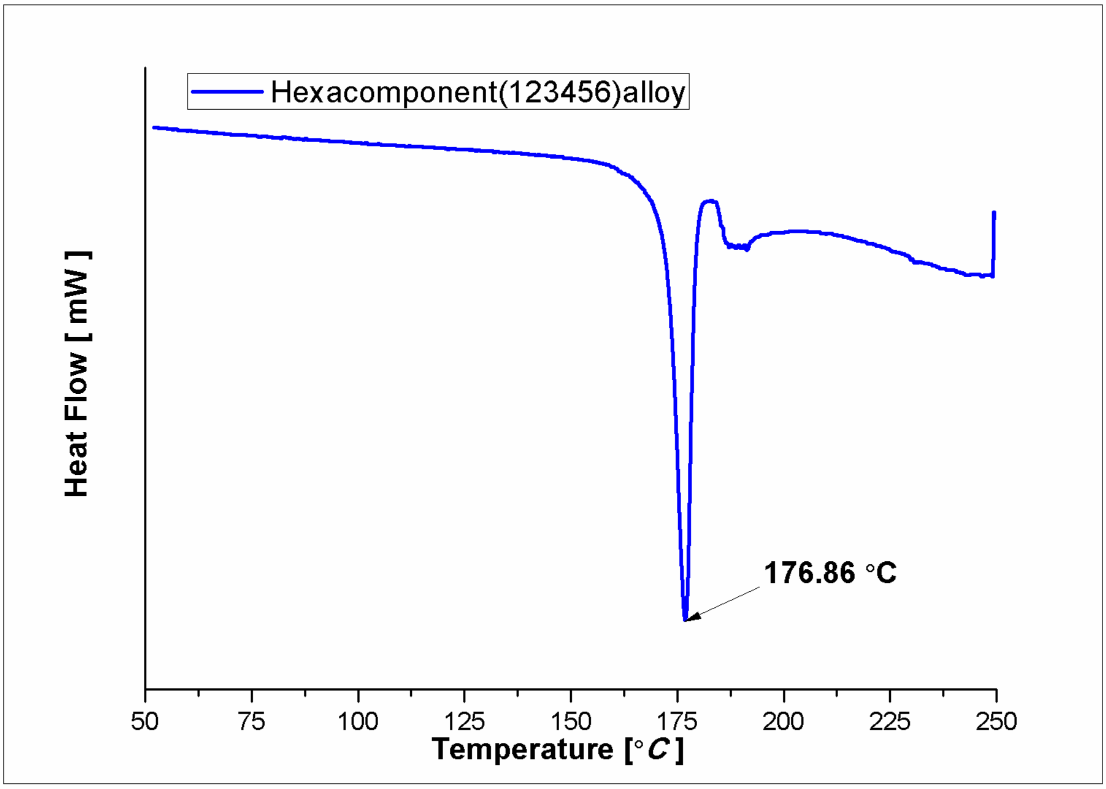

Figure S5b DSC thermogram of Hexacomponent alloy.

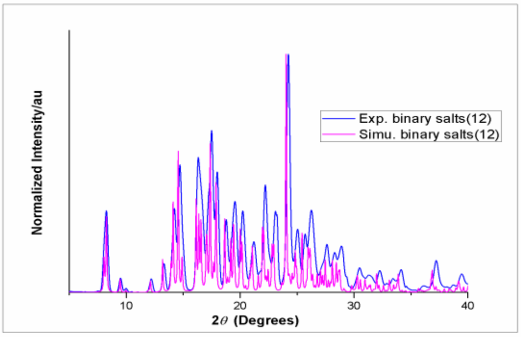

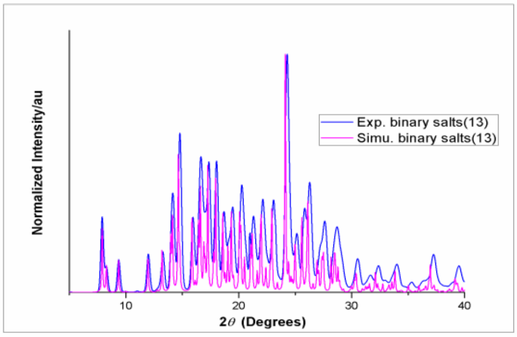

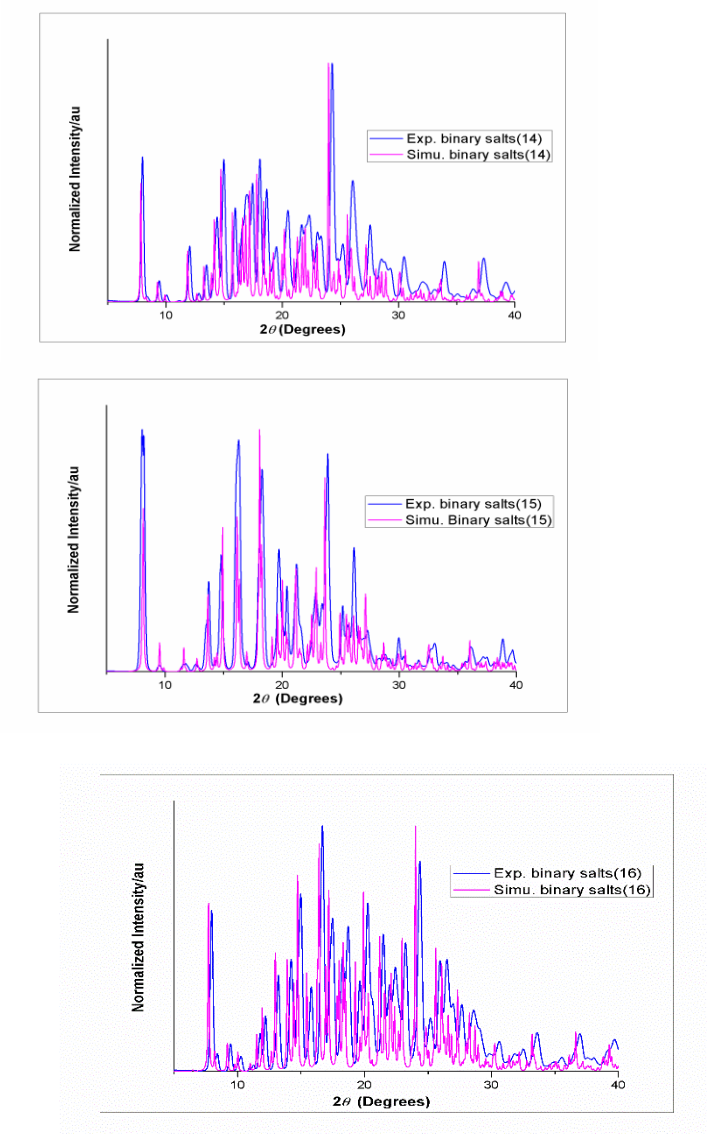

Figure S6a PXRD patterns of Binary salts: Blue indicates experimental PXRD pattern and pink indicates the calculated pattern based on the single crystal X-ray diffraction data.

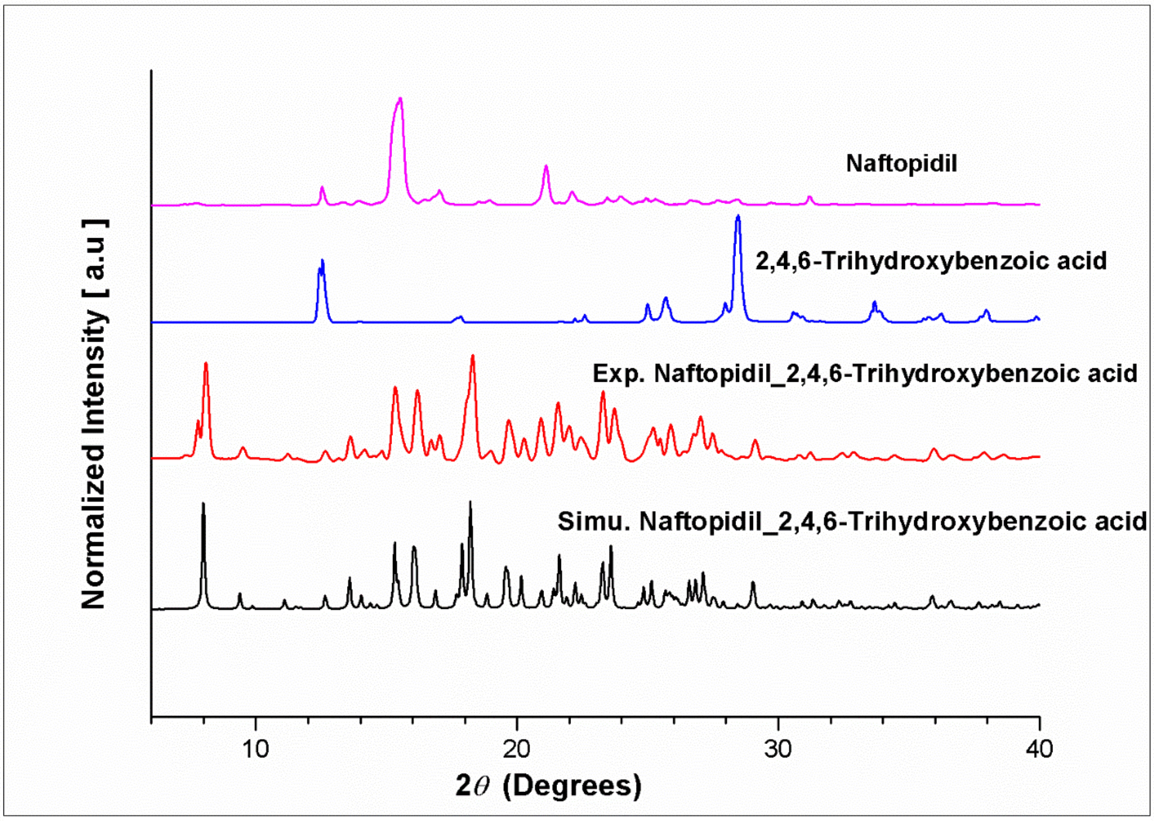

Figure S6b PXRD patterns of Binary salt of Naftopidil and 2,4,6-trihydroxybenzoic acid.

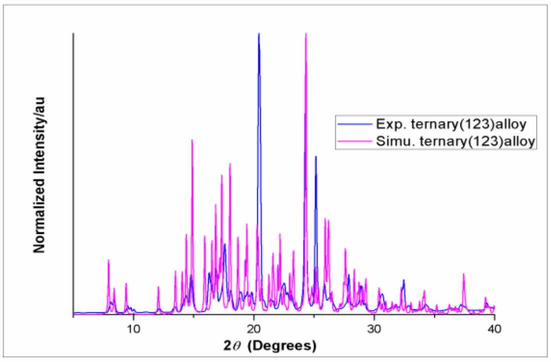
Exp. ternary(123)alloy
Simu. ternary(123)alloy
Normalized Intensity/au
2θ (Degrees)

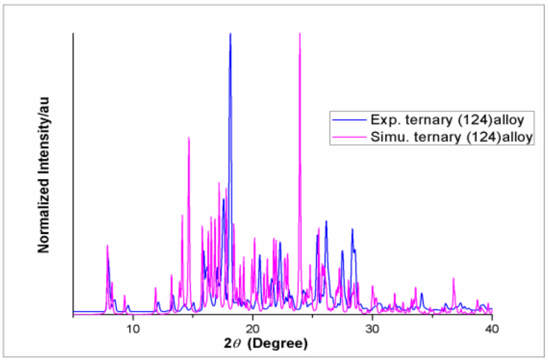
Exp. ternary (124)alloy
Simu. ternary (124)alloy
Normalized Intensity/au
2θ (Degree)

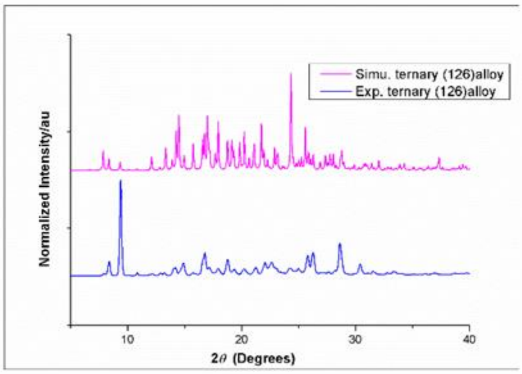
Simu. ternary (126)alloy
Exp. ternary (126)alloy
Normalized Intensity/au
2θ (Degrees)

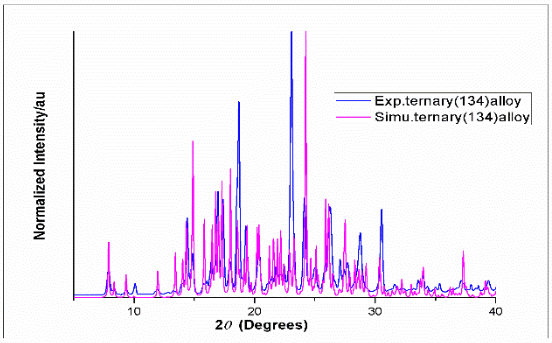
Exp.ternary(134)alloy
Simu.ternary(134)alloy
Normalized Intensity/au
2θ (Degrees)

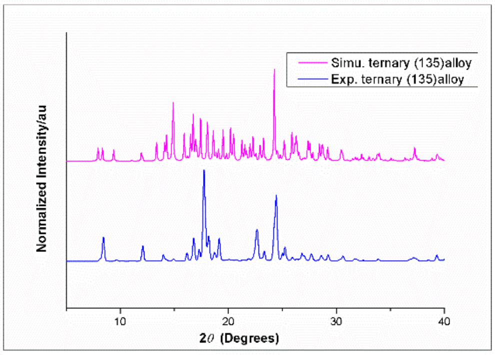
Simu. ternary (135)alloy
Exp. ternary (135)alloy
Normalized Intensity/au
2θ (Degrees)

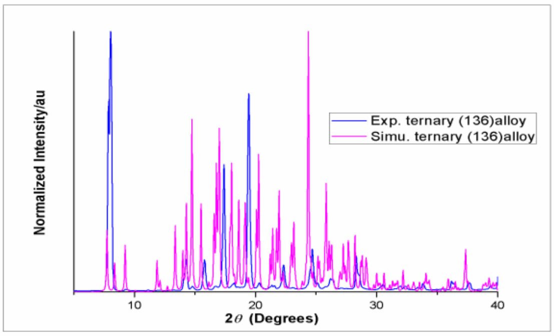
Exp. ternary (136)alloy
Simu. ternary (136)alloy
Normalized Intensity/au
2θ (Degrees)

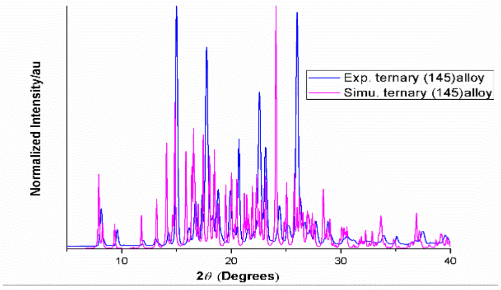
Exp. ternary (145)alloy
Simu. ternary (145)alloy
Normalized Intensity/au
2θ (Degrees)

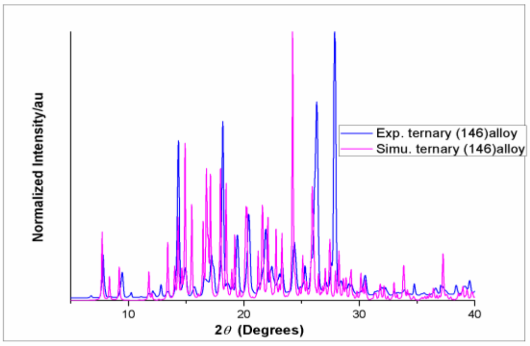
Exp. ternary (146)alloy
Simu. ternary (146)alloy
Normalized Intensity/au
2θ (Degrees)

Figure S7a PXRD patterns of ternary organic salt alloys: Blue indicates experimental PXRD pattern and pink indicates the simulated pattern based on the single crystal X-ray diffraction data.

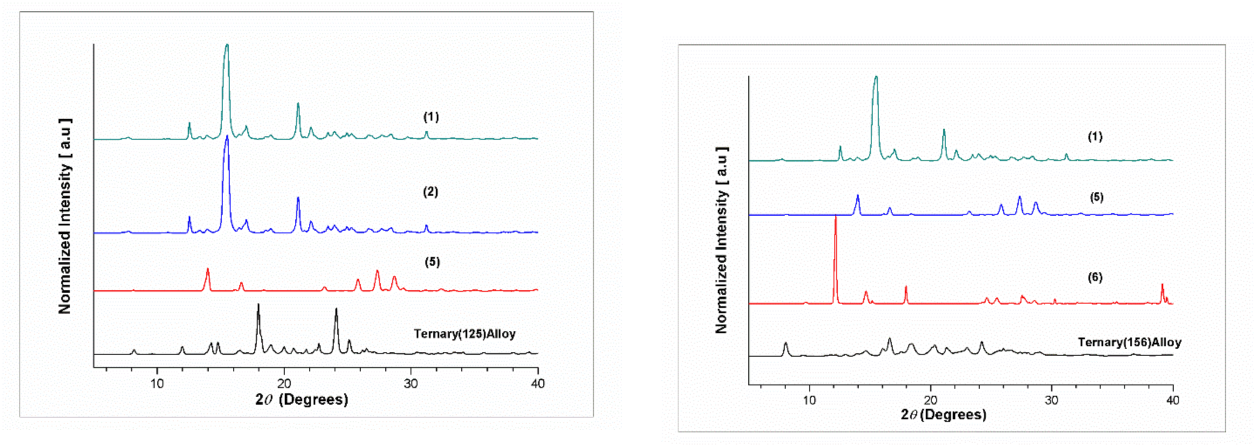

Figure S7b PXRD patterns of 125 and 156 ternary organic salt alloys: Naftopidil (1) and aromatic acids PXRD pattern are compared with corresponding ternary slat alloys.

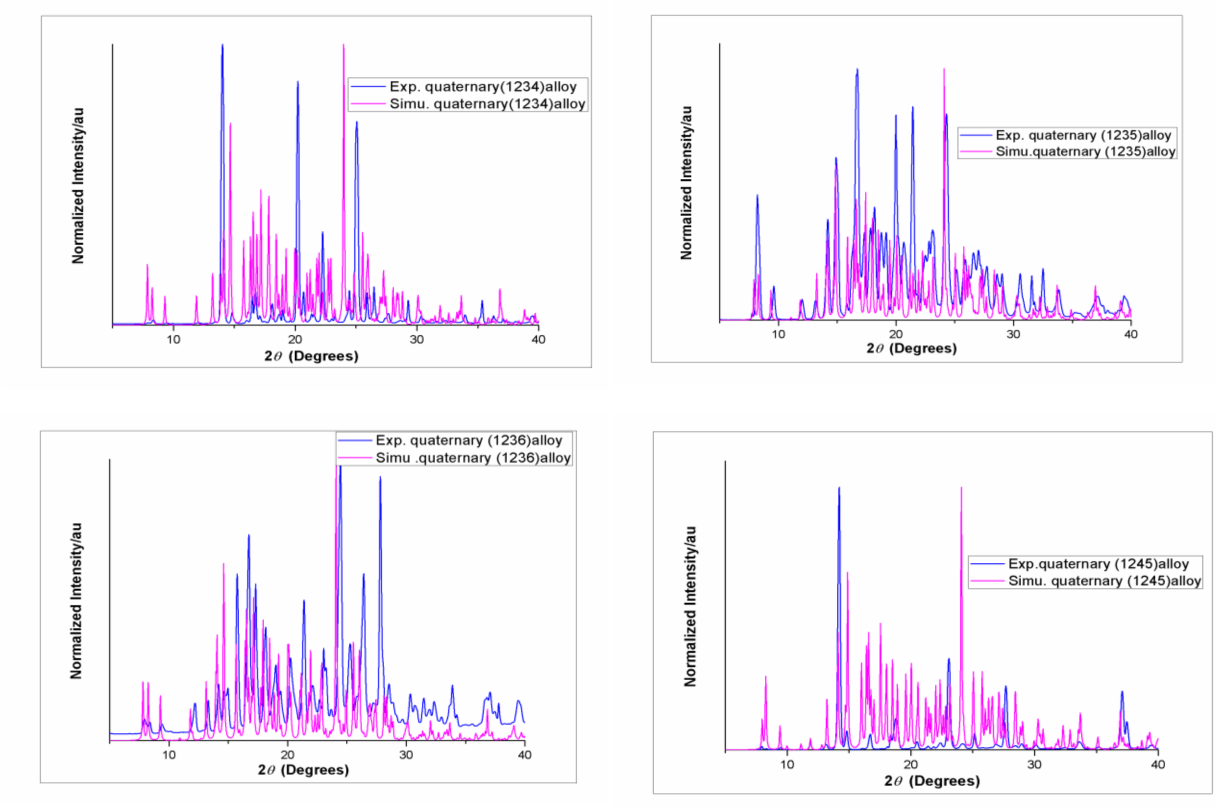

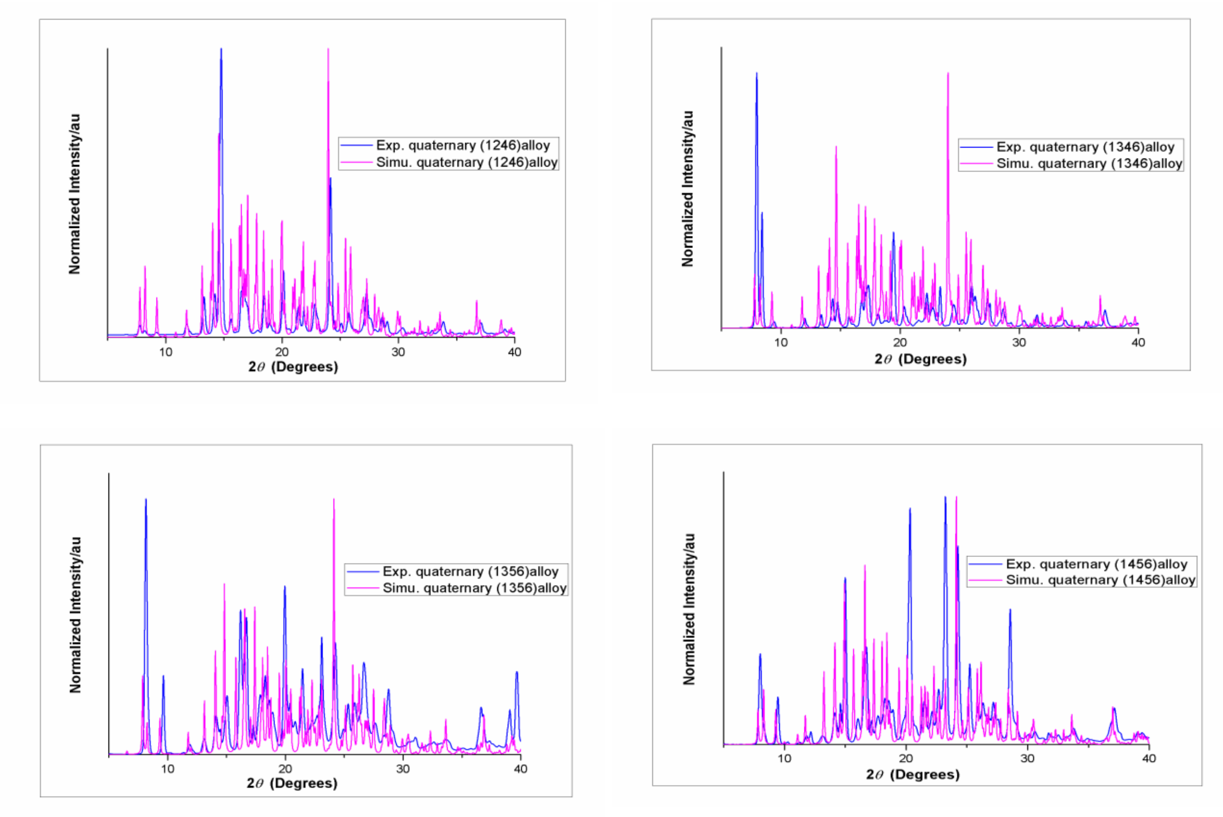

Figure S8a PXRD patterns of quaternary organic salt alloys: Blue indicates experimental PXRD pattern and pink indicates the simulated pattern based on the single crystal X-ray diffraction data.

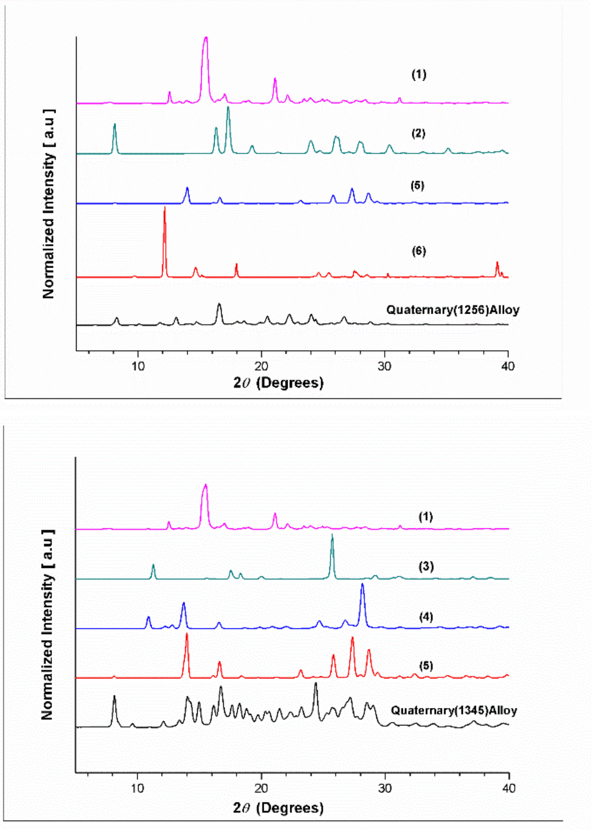

Figure S8b PXRD patterns of 1256 and 1345 quaternary organic salt alloys. Naftopidil (1) and acids PXRD pattern are compared with corresponding quaternary slat alloys.

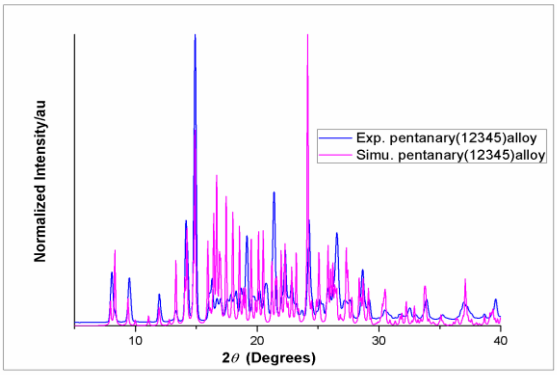

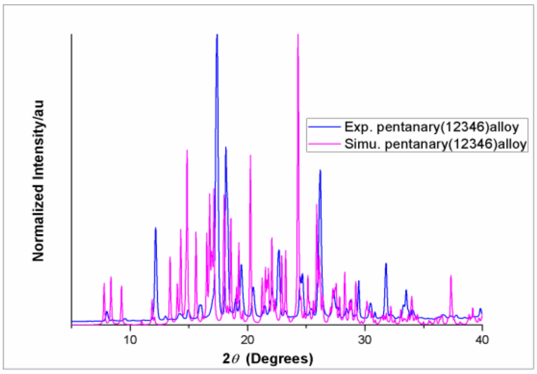

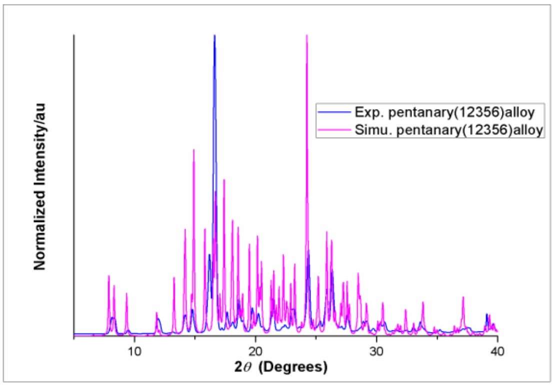

Figure S9a PXRD patterns of pentanary organic salt alloys: Blue indicates experimental PXRD pattern and pink indicates the simulated based on the single crystal X-ray diffraction data.

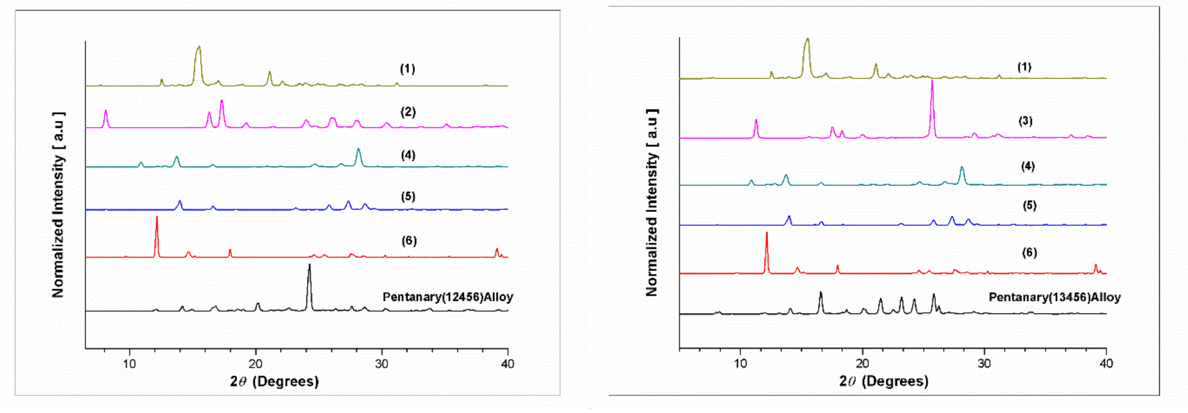

Figure S9b PXRD patterns of 12456 and 13456 pentanary organic salt alloys. Naftopidil (1) and acids PXRD pattern are compared with corresponding pentanary slat alloys.

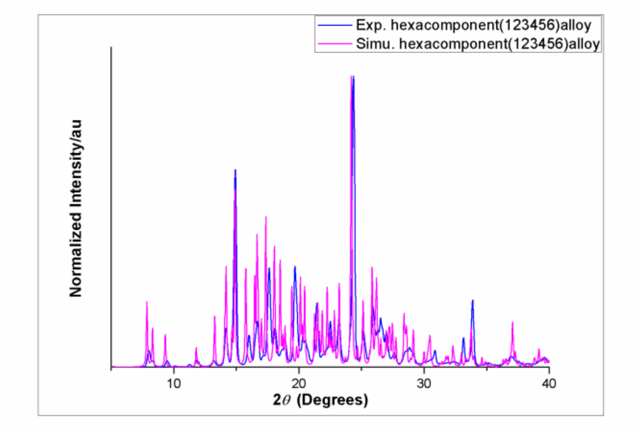

Figure S10 PXRD patterns of hexanary organic salt alloys: Blue indicates experimental PXRD pattern and pink indicates the simulated based on the single crystal X-ray diffraction data.

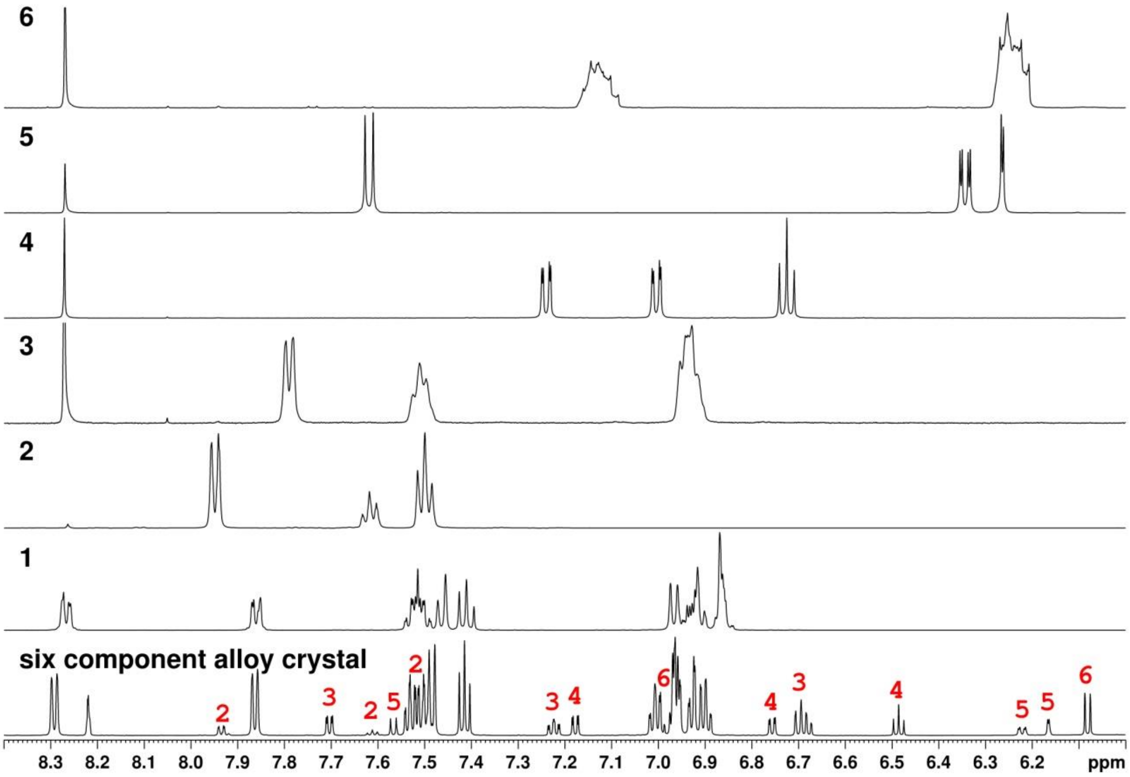

Figure S11 Stacked ^{1}H NMR plot of hexacomponent (123456) salt alloy crystal along with the five carboxylic acid units (2-6, indicated in black font) and the drug (1). For the sake of clarity, only the expanded region between 6-8.4 ppm is shown, in which almost all of the resonances from the carboxylic acid components are populated. The resonances corresponding to the individual carboxylic acid components in the spectrum of the hexanaray crystal are indicated in red font.

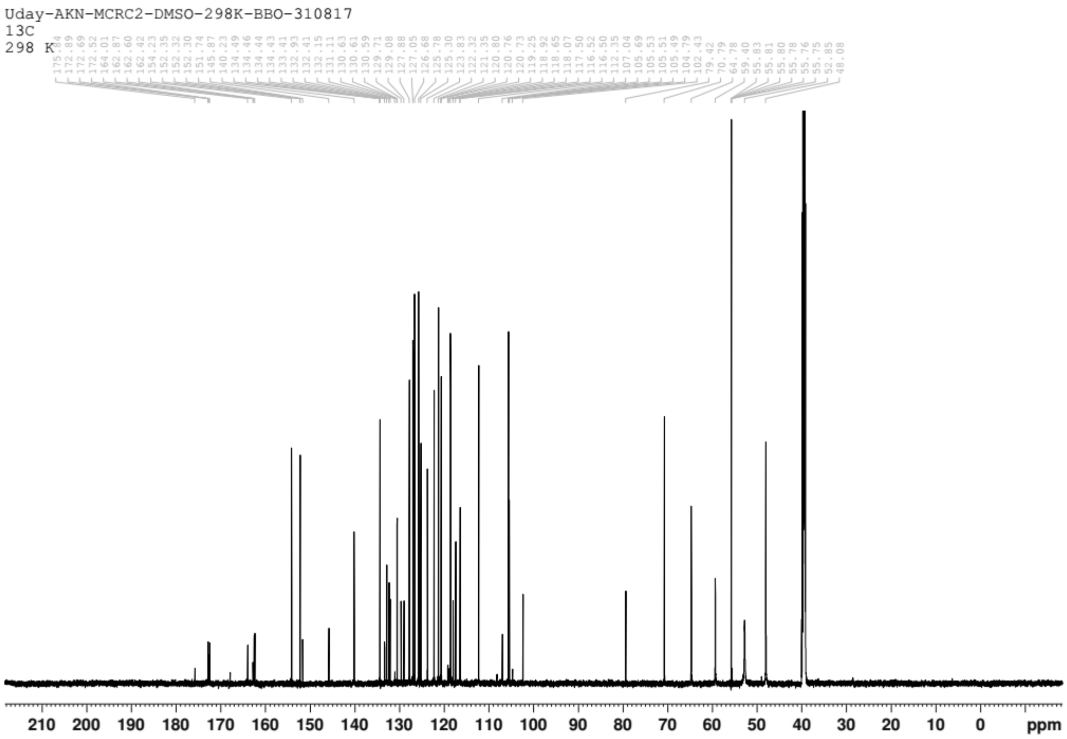

Figure S12 ^{13}C NMR plot of the hexacomponent (123456) alloy crystal.

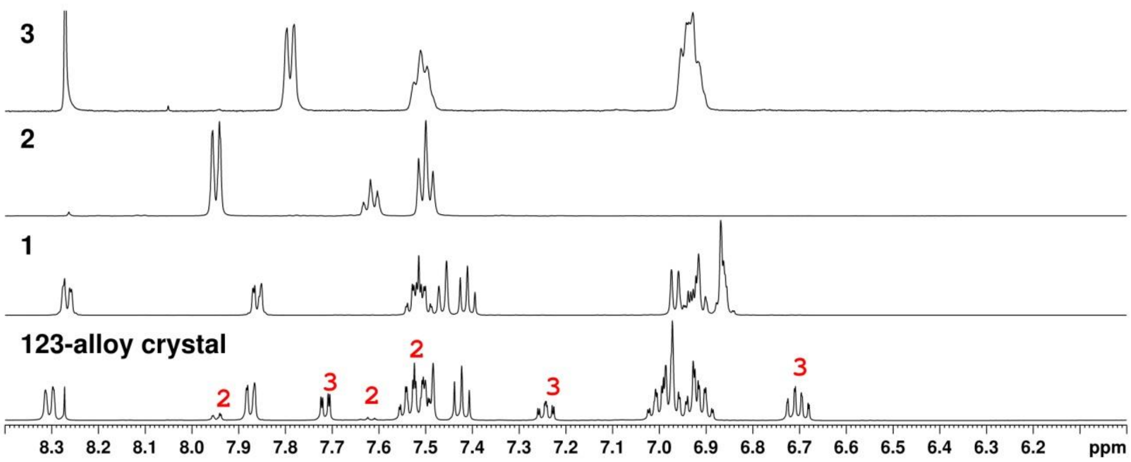

Figure S13 Stacked ^{1}H NMR plot of ternary (123) alloy crystal along with the two carboxylic acid units (2-3, indicated in black font) and the drug (1). The resonances corresponding to the individual carboxylic acid components in the spectrum of the alloy crystal are indicated in red font.

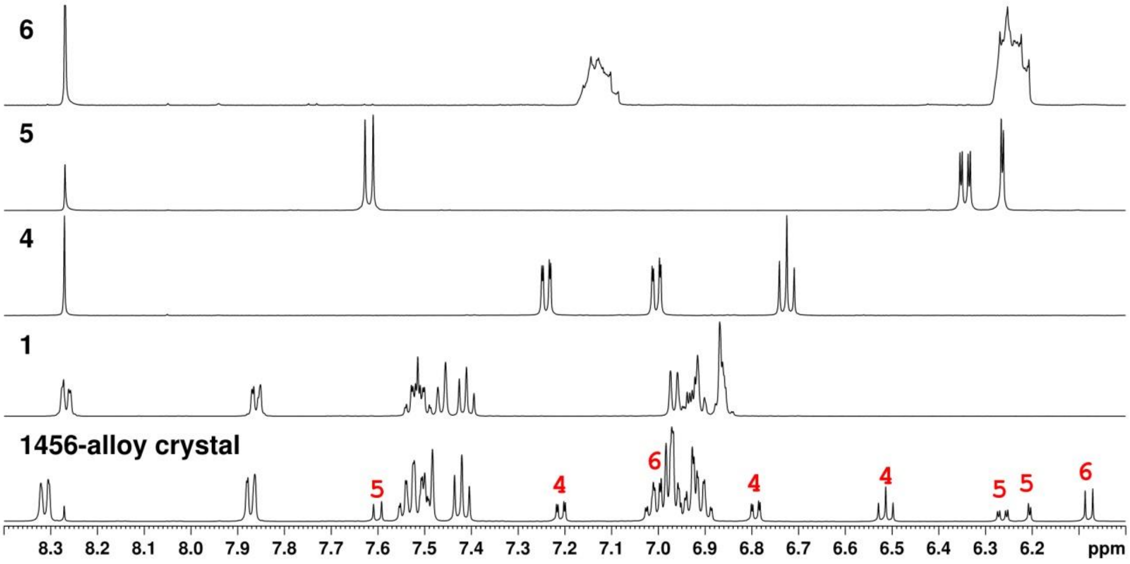

Figure S14 Stacked ^{1}H NMR plot of quaternary (1456) alloy crystal along with the three carboxylic acid units (4-6, indicated in black font) and the drug (1). The resonances corresponding to the individual carboxylic acid components in the spectrum of the alloy crystal are indicated in red font.

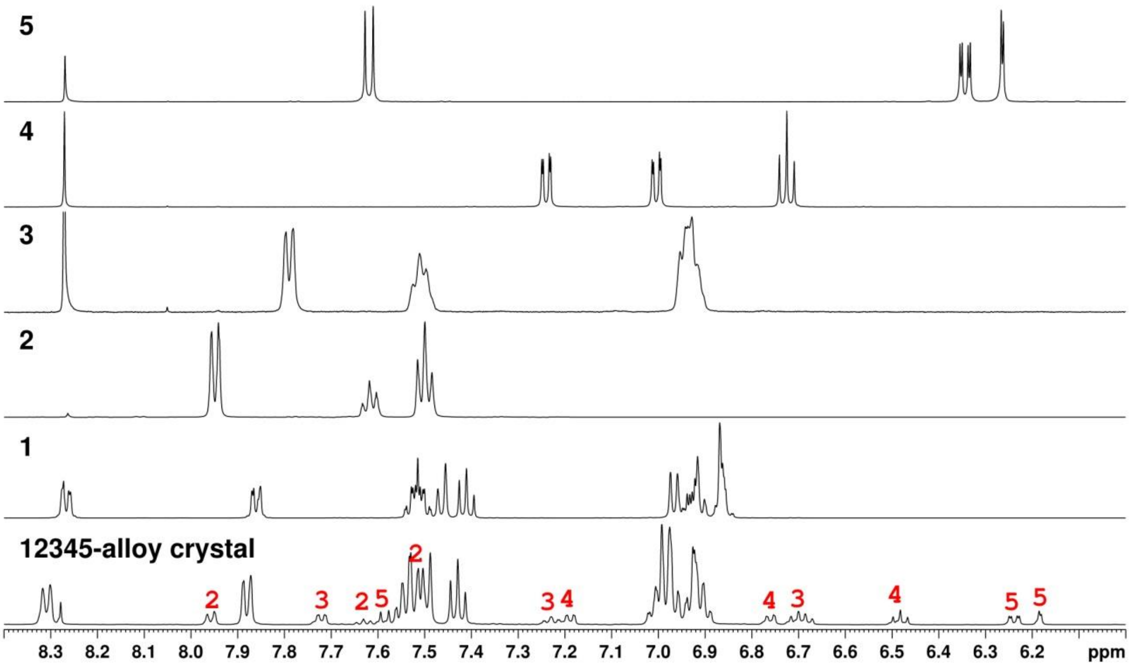

Figure S15 Stacked ^{1}H NMR plot of pentenary (12345) alloy crystal along with the four carboxylic acid units (2-5, indicated in black font) and the drug (1). The resonances corresponding to the individual carboxylic acid components in the spectrum of the alloy crystal are indicated in red font.

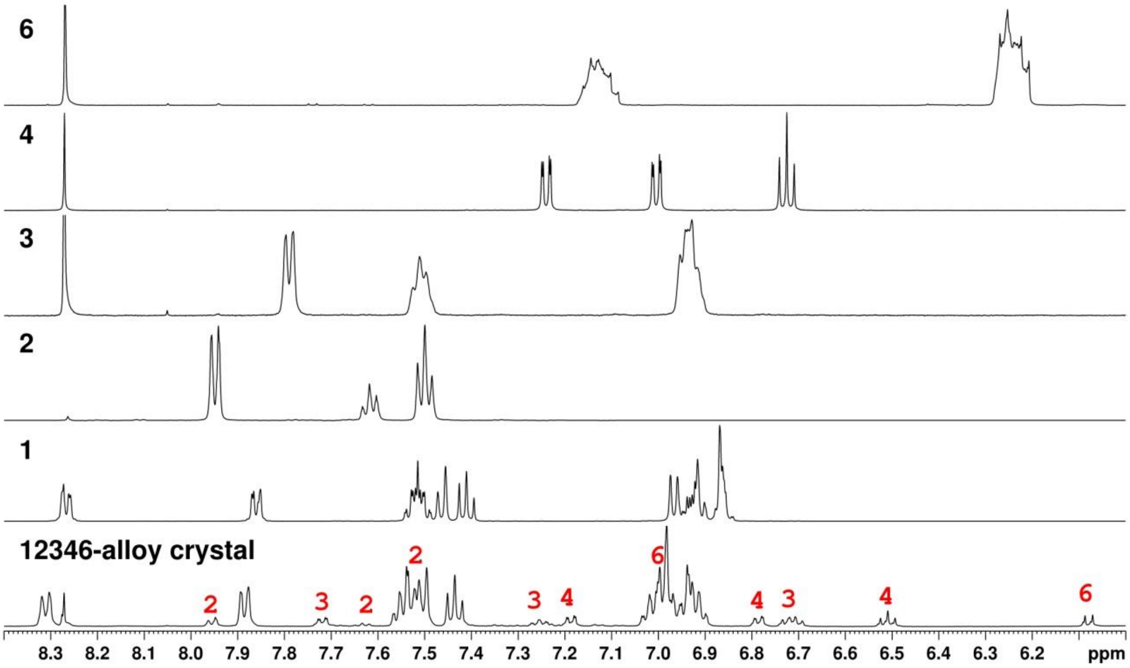

Figure S16 Stacked ^{1}H NMR plot of pentenary (12346) alloy crystal along with the four carboxylic acid units (2-4, 6, indicated in black font) and the drug (1). The resonances corresponding to the individual carboxylic acid components in the spectrum of the alloy crystal are indicated in red font.

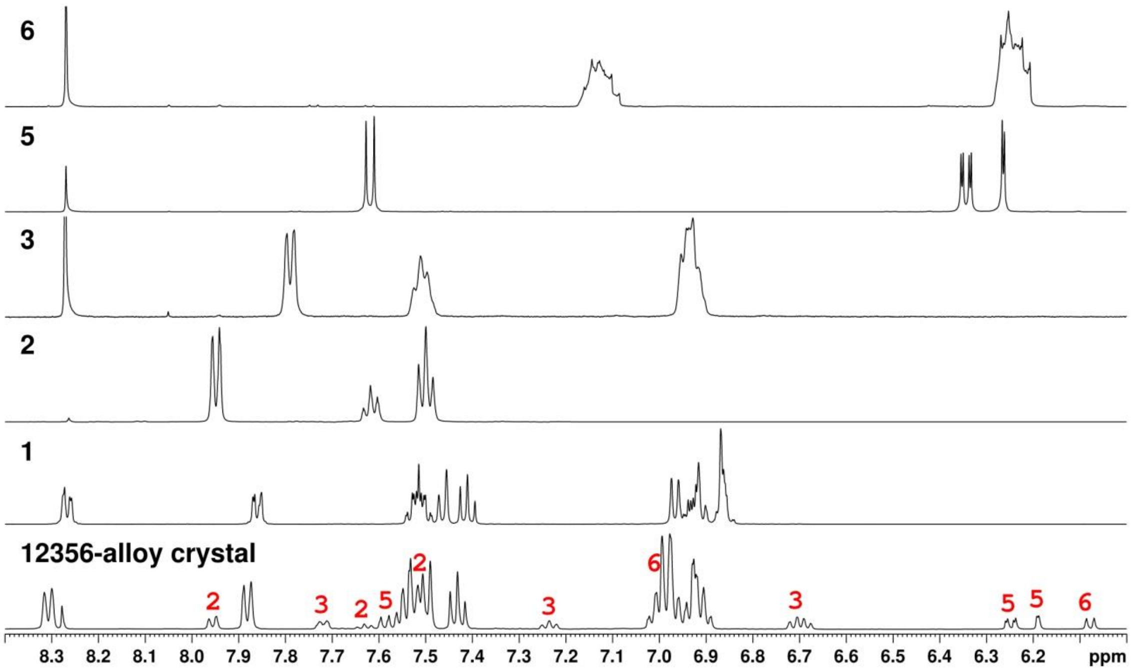

Figure S17 Stacked ^{1}H NMR plot of pentenary (12356) alloy crystal along with the four carboxylic acid units (2-3, 5-6 indicated in black font) and the drug (1). The resonances corresponding to the individual carboxylic acid components in the spectrum of the alloy crystal are indicated in red font.

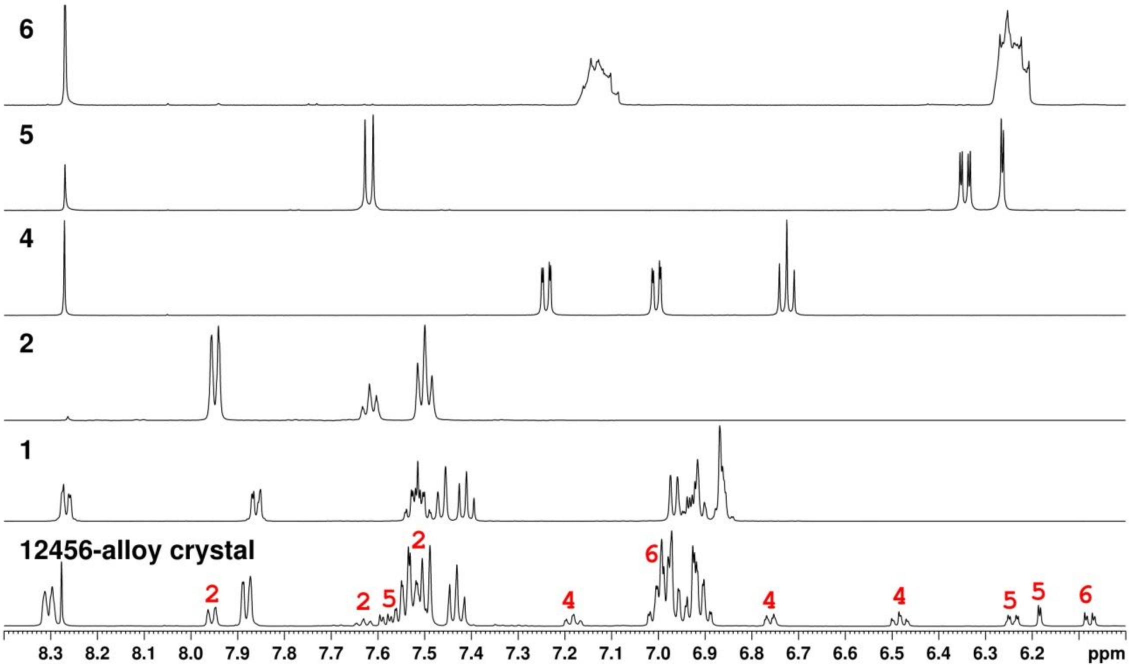

Figure S18 Stacked ^{1}H NMR plot of pentenary (12456) alloy crystal along with the four carboxylic acid units (2, 4-6, indicated in black font) and the drug (1). The resonances corresponding to the individual carboxylic acid components in the spectrum of the alloy crystal are indicated in red font.

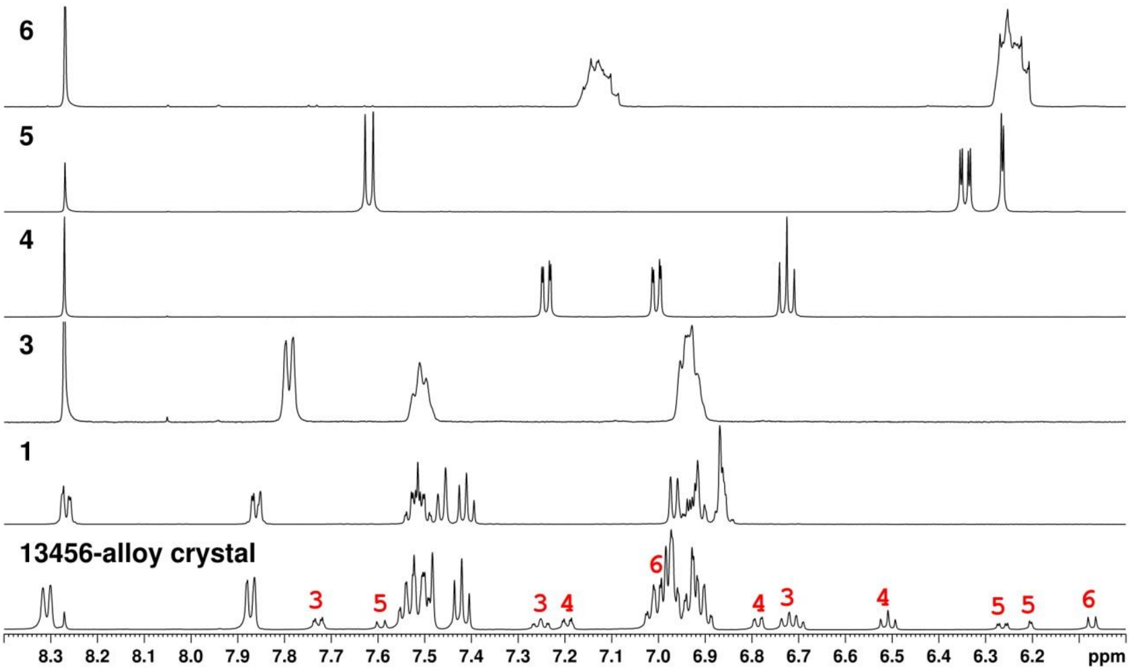

Figure S19 Stacked ^{1}H NMR plot of pentenary (13456) alloy crystal along with the four carboxylic acid units (3-6, indicated in black font) and the drug (1). The resonances corresponding to the individual carboxylic acid components in the spectrum of the alloy crystal are indicated in red font.

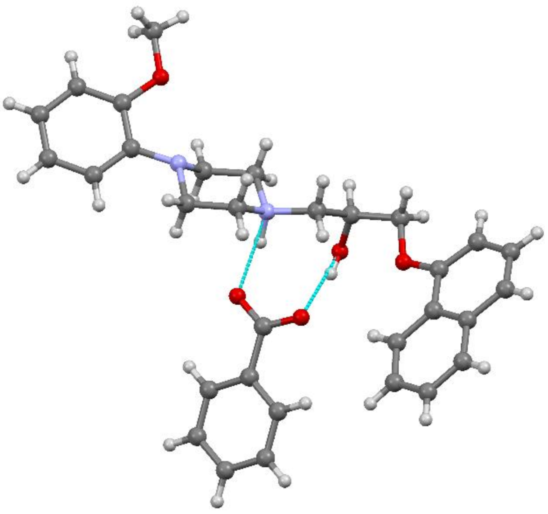

Figure S20 Binary salt (12).

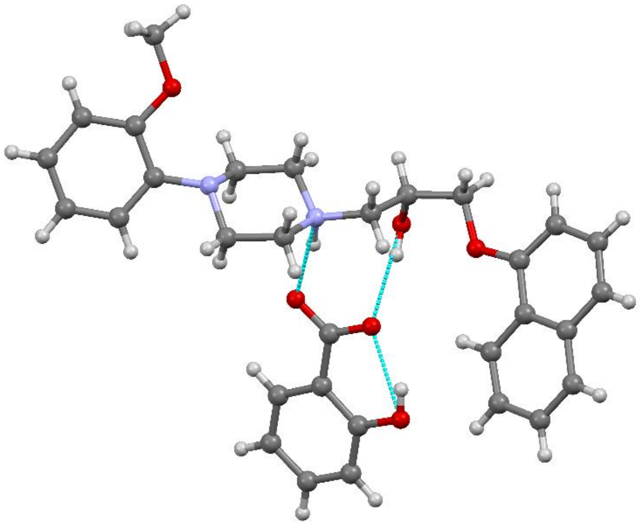

Figure S21 Binary salt (13).

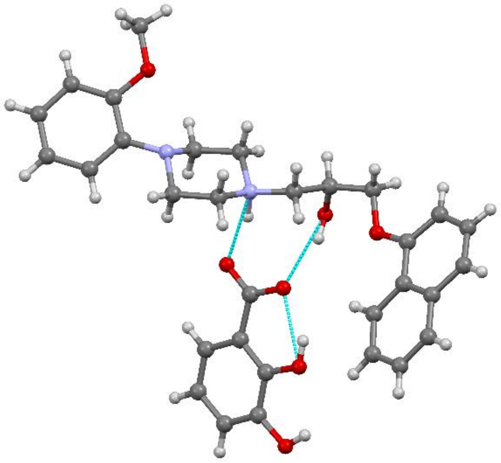

Figure S22 Binary salt (14).

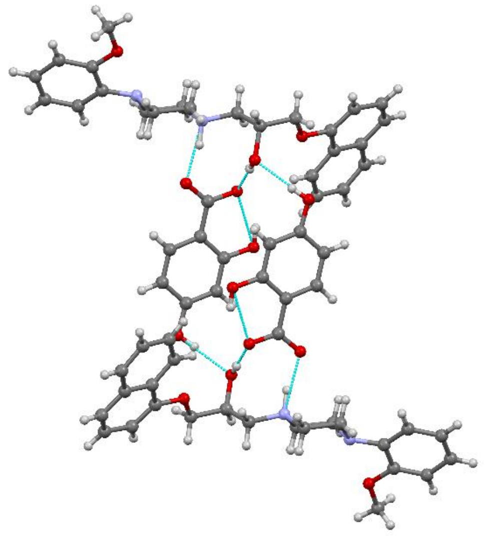

Figure S23 Binary salt (15).

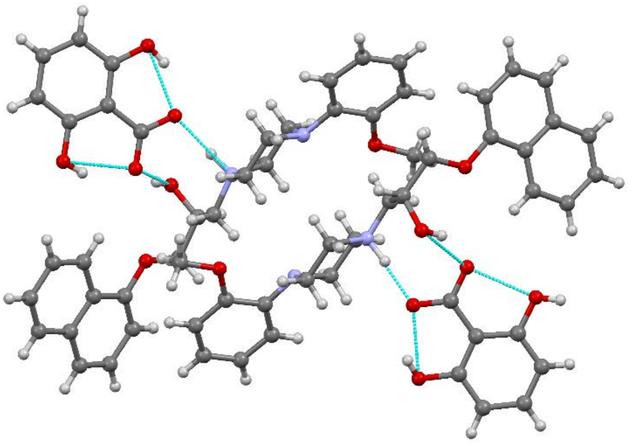

Figure S24 Binary salt (16).

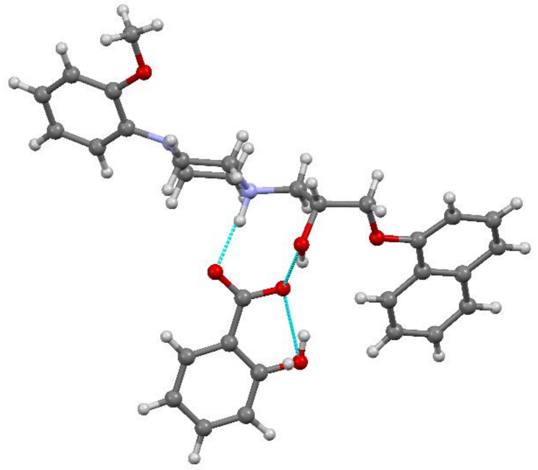

Figure S25 Organic alloy (123).

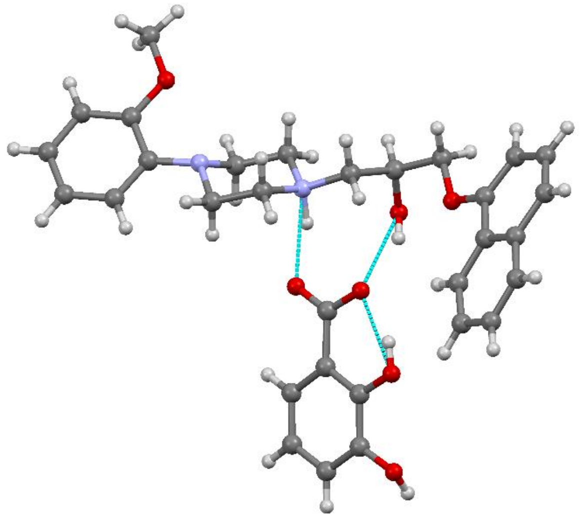

Figure S26 Organic alloy (124).

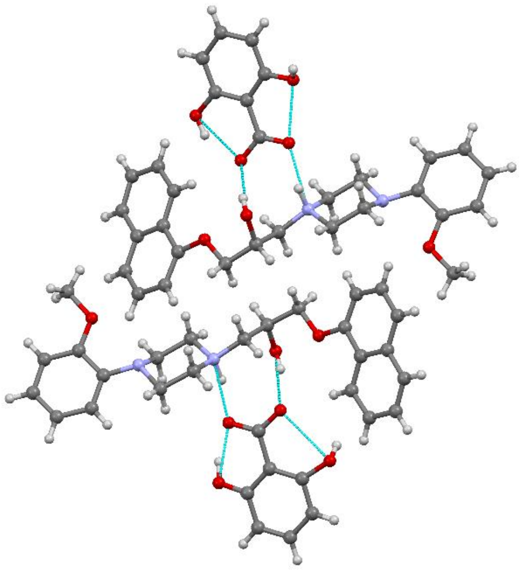

Figure S27 Organic alloy (126).

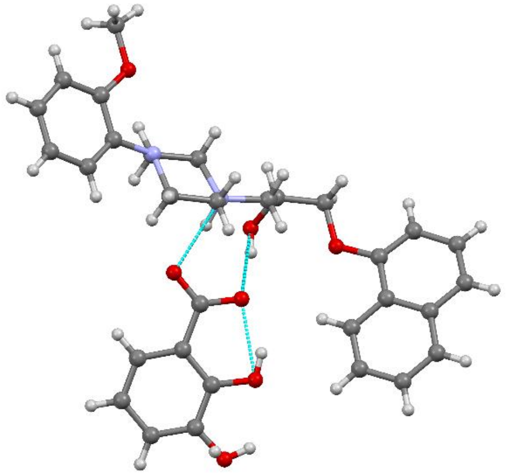

Figure S28 Organic alloy (134).

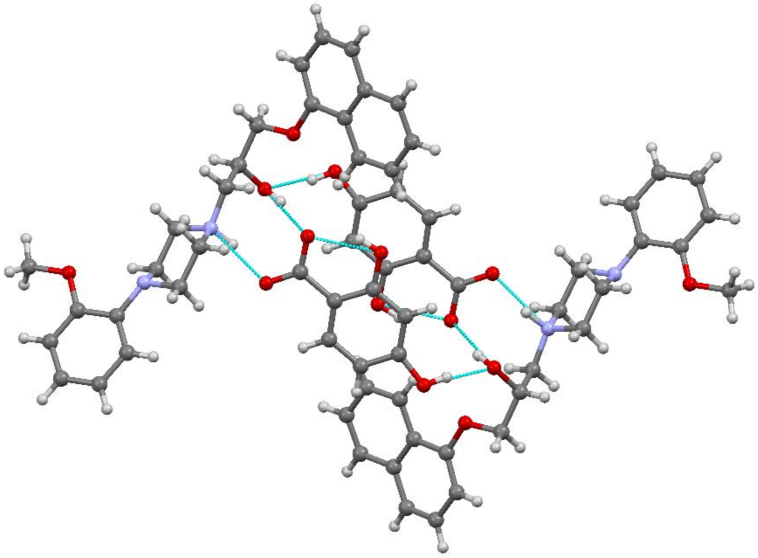

Figure S29 Organic alloy (135).

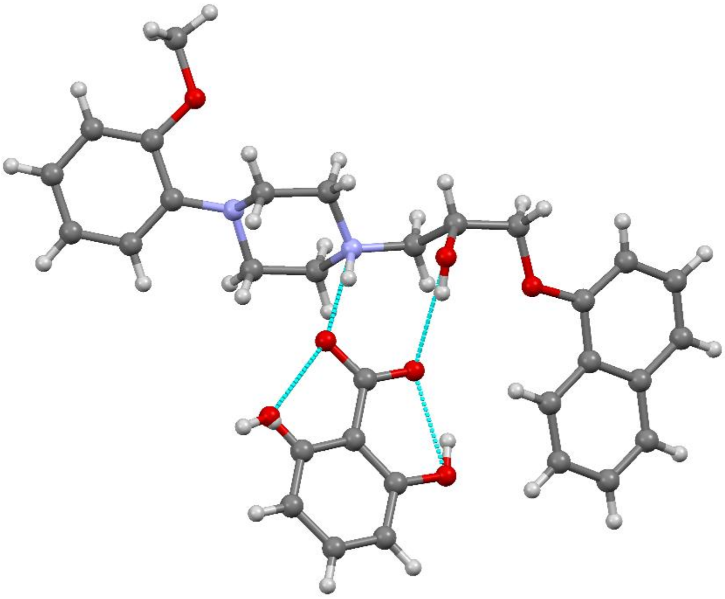

Figure S30 Organic alloy (136).

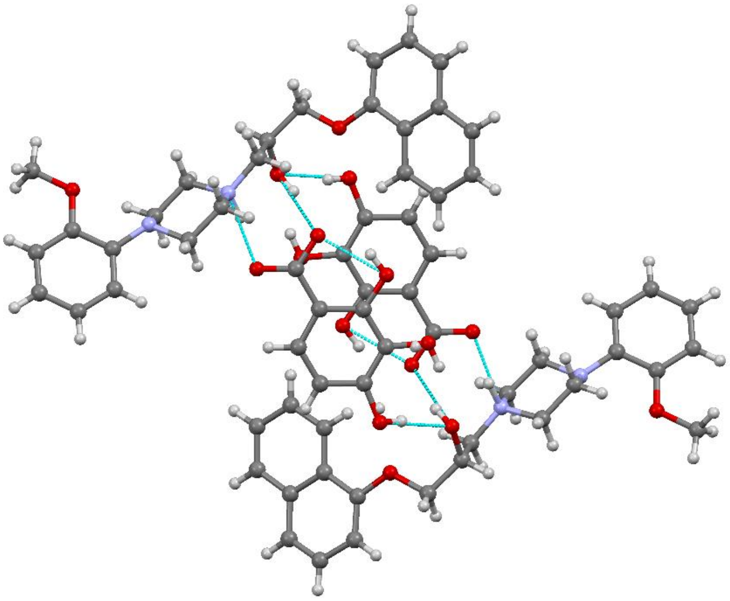

Figure S31 Organic alloy (145).

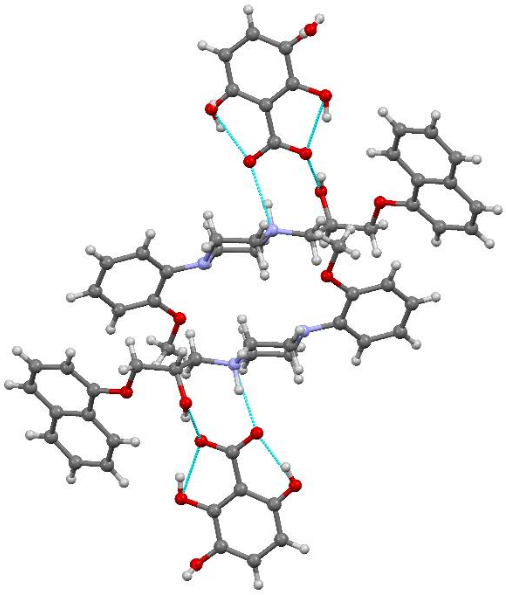

Figure S32 Organic alloy (146).

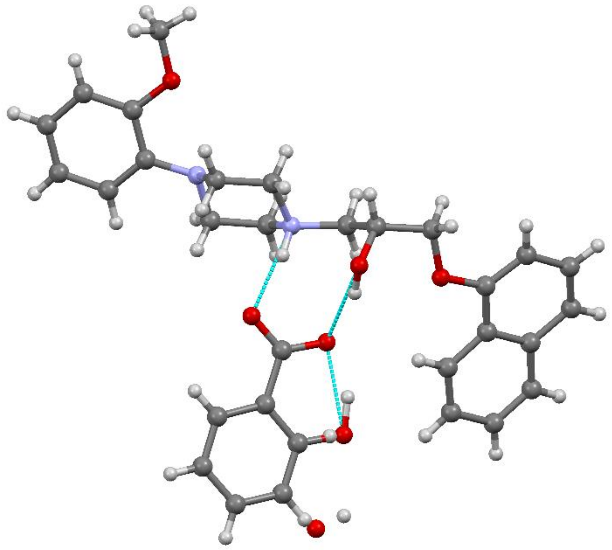

Figure S33 Organic alloy (1234).

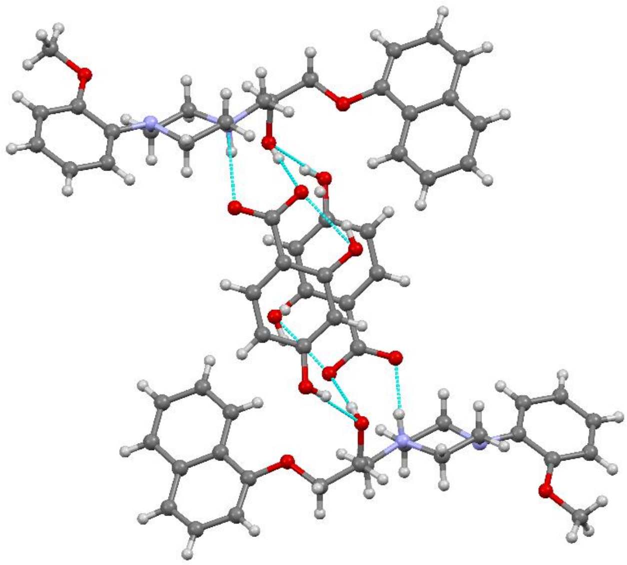

Figure S34 Organic alloy (1235).

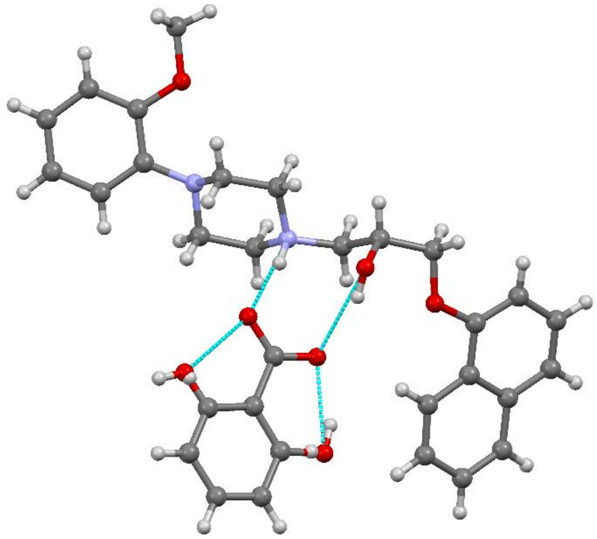

Figure S35 Organic alloy (1236).

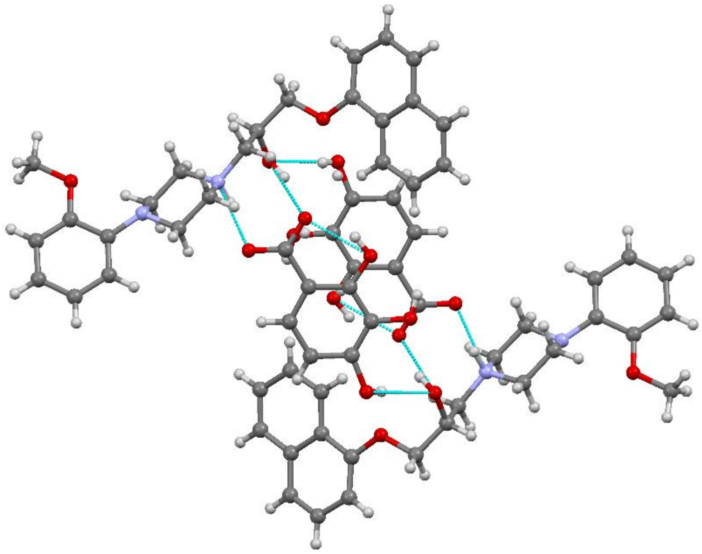

Figure S36 Organic alloy (1245).

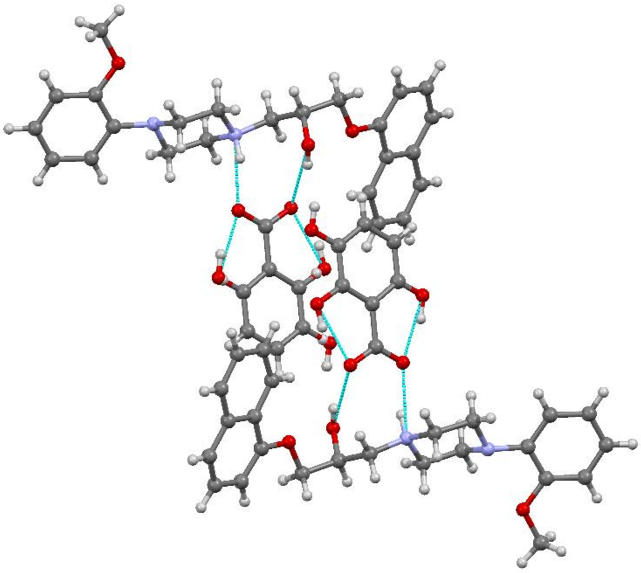

Figure S37 Organic alloy (1246).

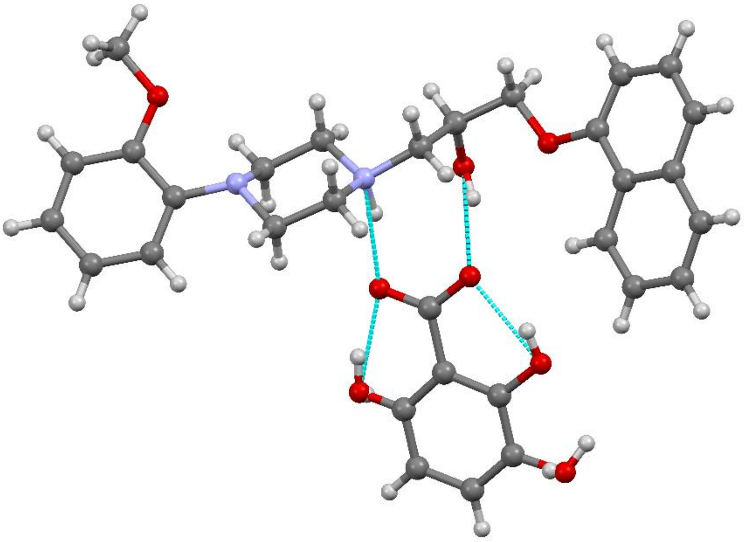

Figure S38 Organic alloy (1346).

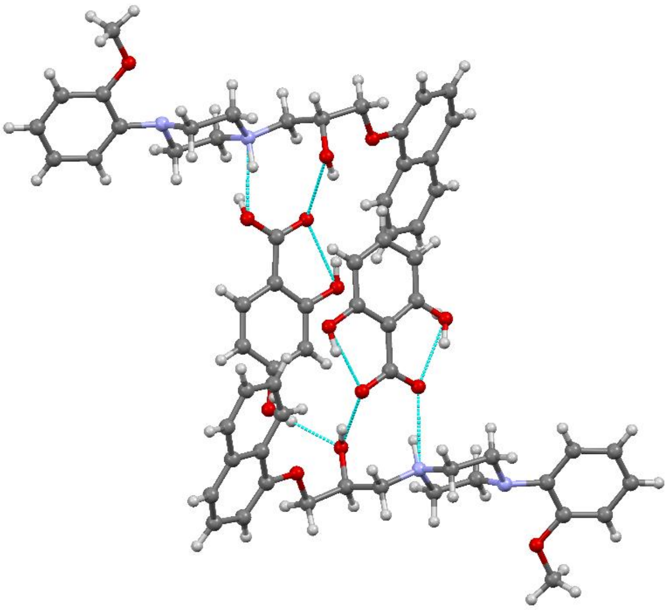

Figure S39 Organic alloy (1356).

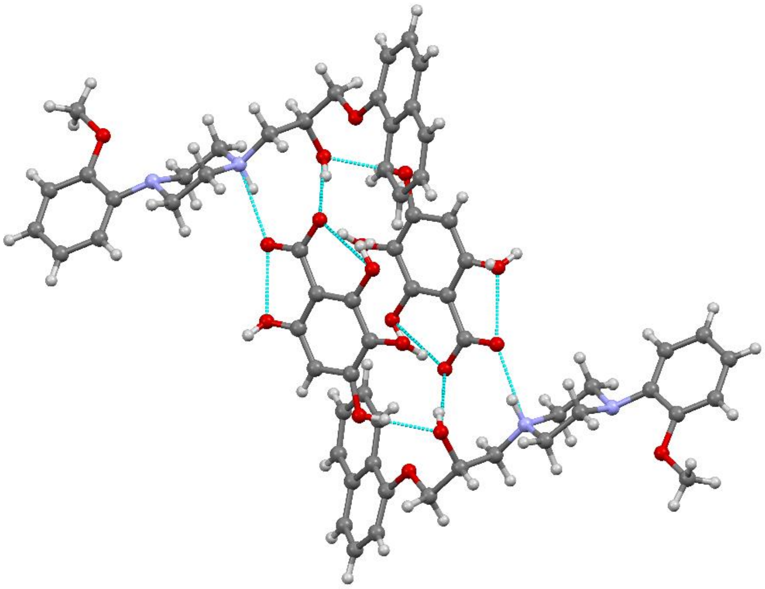

Figure S40 Organic alloy (1456).

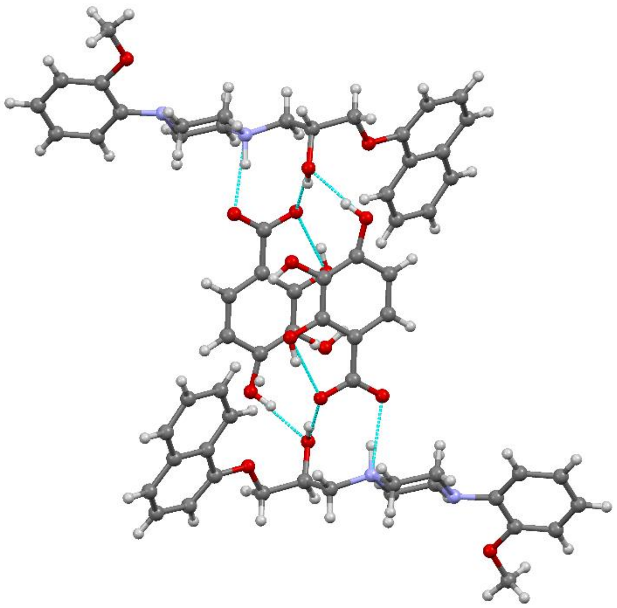

Figure S41 Organic alloy (12345).

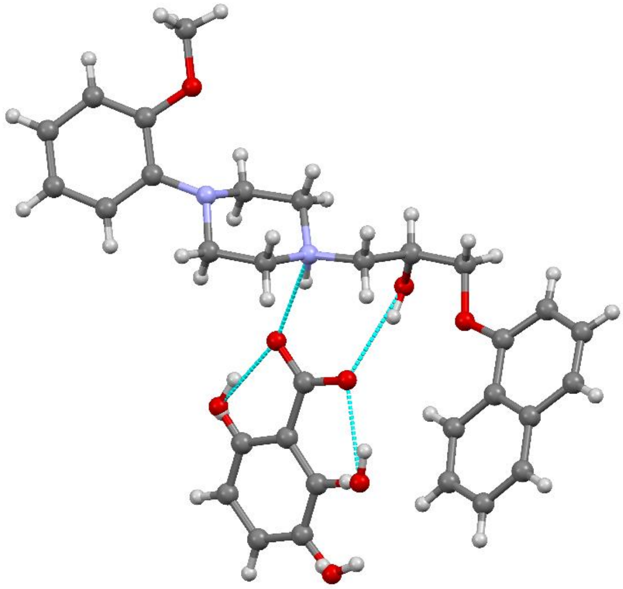

Figure S42 Organic alloy (12346).

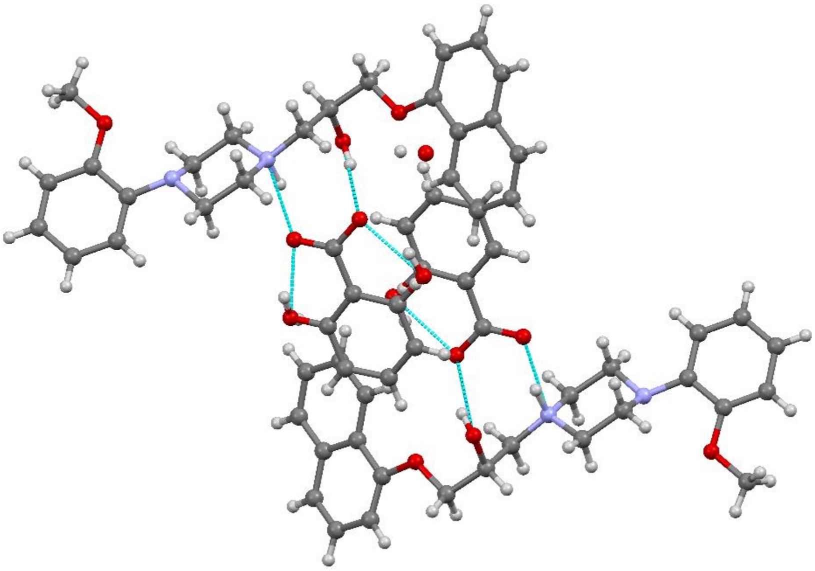

Figure S43 Organic alloy (12356).

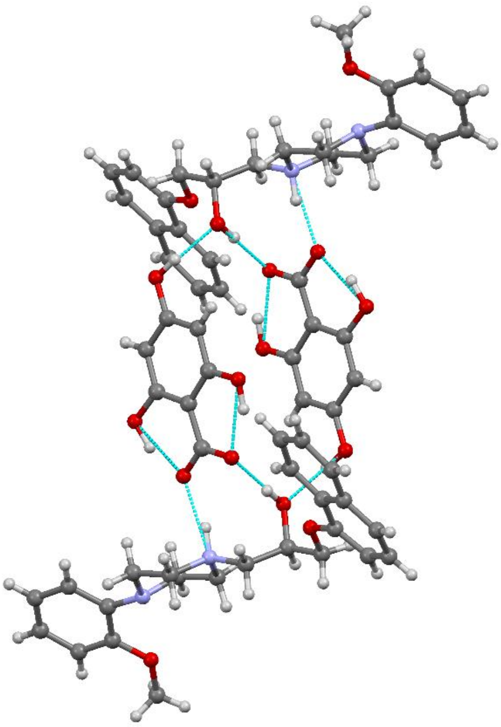

Figure S44 Binary slat of Naftopidil-2,4,6-trihydroxy benzoic acid.
